# Impact of the solvent capacity constraint on *E. coli *metabolism

**DOI:** 10.1186/1752-0509-2-7

**Published:** 2008-01-23

**Authors:** Alexei Vazquez, Qasim K Beg, Marcio A deMenezes, Jason Ernst, Ziv Bar-Joseph, Albert-László Barabási, László G Boros, Zoltán N Oltvai

**Affiliations:** 1 The Simons Center for Systems Biology, Institute for Advanced Study, Princeton, NJ 08540, USA; 2 Department of Pathology, University of Pittsburgh, Pittsburgh, PA, 15261, USA; 3 Instituto de Física, Universidade Federal Fluminense, Rio de Janeiro, 24210, Brazil; 4 Machine Learning Department, Carnegie-Mellon University, Pittsburgh, PA, 15217, USA; 5 Department of Physics and Center for Complex Networks Research, University of Notre Dame, South Bend, IN 46556, USA; 6 SiDMAP, LLC and the UCLA School of Medicine, Los Angeles, CA 90064, USA; 7 Department of Biomedical Engineering, Boston University, Boston, MA 02215, USA

## Abstract

**Background:**

Obtaining quantitative predictions for cellular metabolic activities requires the identification and modeling of the physicochemical constraints that are relevant at physiological growth conditions. Molecular crowding in a cell's cytoplasm is one such potential constraint, as it limits the solvent capacity available to metabolic enzymes.

**Results:**

Using a recently introduced flux balance modeling framework (FBAwMC) here we demonstrate that this constraint determines a metabolic switch in *E. coli *cells when they are shifted from low to high growth rates. The switch is characterized by a change in effective optimization strategy, the excretion of acetate at high growth rates, and a global reorganization of *E. coli *metabolic fluxes, the latter being partially confirmed by flux measurements of central metabolic reactions.

**Conclusion:**

These results implicate the solvent capacity as an important physiological constraint acting on *E. coli *cells operating at high metabolic rates and for the activation of a metabolic switch when they are shifted from low to high growth rates. The relevance of this constraint in the context of both the aerobic ethanol excretion seen in fast growing yeast cells (Crabtree effect) and the aerobic glycolysis observed in rapidly dividing cancer cells (Warburg effect) should be addressed in the future.

## Background

Understanding an organism's metabolism at a system level requires knowledge of the physicochemical constraints limiting its metabolic capabilities under different growth conditions, and the genetic regulatory mechanisms that ultimately allow it to adapt to a changing environment. In some cases there is an obvious connection between an environmental change and the regulatory mechanisms responding to it, an example being a switch from aerobic to anaerobic growth [1]. However, there are constraints leading to less obvious metabolic changes, involving a complex global rearrangement of the cell's metabolism. A key aim of systems biology is to uncover the metabolic constraints determining such complex phenotypic changes, which can be understood only when the system is analyzed at a global scale [2-4].

In the absence of cell-scale kinetic models, flux balance analysis (FBA) provides experimentally testable predictions on an organism's metabolic flux state [4-8], which are based on conservation principles, particularly mass conservation, and metabolic capacity constraints. The impact of local constraints, such as uptake capacities, have been investigated [4-7], and capacity constraints over full metabolic pathways have been considered as well [9]. Moreover, it has been hypothesized that the high concentration of macromolecules in the cell's cytoplasm imposes a global constraint on the metabolic capacity of an organism [10,11]. More recently, we demonstrated that the key quantity is the total intracellular volume available to metabolic enzymes that result in a limited solvent capacity [12]. The addition of the solvent capacity constraint to a FBA model allowed us to explain, within a metabolic efficiency framework, the hierarchy of substrate consumption of *E. coli *cells growing in a mixture of carbon sources [12]. On the other hand, the pattern of substrate consumption can also be reproduced by superimposing regulatory information obtained e.g., from microarray data [13]. Taking together, these results indicate that the FBA model together with the solvent capacity constraint can be used to predict the regulatory mechanisms and, equally importantly, to understand their advantage in terms of metabolic efficiency and constraints. It is not clear, however, if the limited capacity constraint play a role at other physiological growth conditions, e.g., when nutrients are scarce.

Here we study the impact of the limited solvent capacity on *E. coli *cell metabolism at different physiological growth conditions. We demonstrate that this constraint is relevant for fast growing cells, and predict the existence of a metabolic switch between cells growing at low and high nutrient abundance, respectively. We carry out flux measurements of several reactions in the *E. coli *central metabolism, observing a partial agreement with the model predictions. Moreover, to uncover the regulatory mechanisms that control the changes in flux rates, we perform gene expression and enzyme activity measurements, finding that the switch is controlled predominantly at the enzyme activity level implemented by changes in the activity of a few key enzymes in the *E. coli *central metabolism. Finally, we discuss the potential relevance of the limited solvent capacity constraint to experimental observations in other organisms.

## Results

### Limited solvent capacity constrains the metabolic rate of fast growing *E. coli *cells

The cell's cytoplasm is characterized by a high concentration of macromolecules [14] resulting in a limited solvent capacity for the allocation of metabolic enzymes. More precisely, given that the enzyme molecules have a finite molar volume *v*_*i *_only a finite number of them fit in a given cell volume *V*. Indeed, if *n*_*i *_is the number of moles of the *i*^th ^enzyme, then

$$\sum_{i=1}^{N}v_{i}n_{i}\leq V,$$

where the inequality sign accounts for the volume of other cell components and the free volume necessary for cellular transport as well. Dividing by cell mass *M *we can reformulate this inequality in terms of the enzyme concentrations *E*_*i *_= *n*_*i*_/*M *(moles/unit mass), resulting in

$$\sum_{i=1}^{N}v_{i}E_{i}\leq \frac{1}{C},$$

where *C *= *M*/*V *is the cytoplasmic density. An enzyme concentration *E*_*i *_results in a flux *f*_*i *_= *b*_*i*_*E*_*i *_over reaction *i*, where the parameter *b*_*i *_is determined by the reaction mechanism, kinetic parameters, and metabolite concentrations. Therefore, the enzyme concentration constraint (Eq. 2) is reflected in the metabolic flux constraint

$$\sum_{i=1}^{N}a_{i}f_{i}\leq 1,$$

where

$$a_{i}=\frac{Cv_{i}}{b_{i}}.$$

Since the coefficients *a*_*i *_(units of inverse flux) quantifies the contribution to the overall crowding by reaction *i *we refer to them as the 'crowding coefficients'.

**Figure 1:**
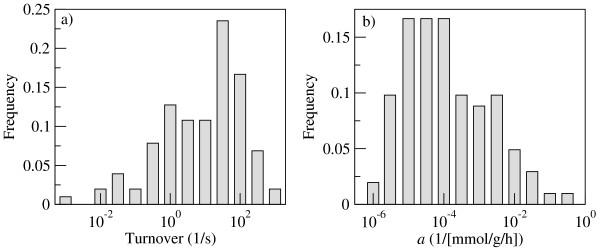
**Estimating the crowding coefficients of *E. coli *metabolic enzymes**: (a) Distribution of turnover rates of *E. coli *enzymes as obtained from the BRENDA data base [17]; (b) Distribution of crowding coefficients among a hundred *E. coli *enzymes, as obtained using Eq. 4.

To understand the relevance of the constraint (Eq. 3) at physiological growth conditions we first estimate the crowding coefficients (Eq. 4) using data from experimental reports. The *E. coli *cytoplasmic density of macromolecules is *C *= 0.34 g/ml [15], while the molar volumes of proteins are proportional to their molar masses [16]. The coefficient of proportionality represents the specific volume and it is about 0.73 ml/g. This empirical law allows us to compute the molar volumes of *E. coli *enzymes from their molar masses. As a first approximation we estimate *b*_*i*_, the coefficient of proportionality between reaction rate and enzyme concentration, from the enzyme's turnover numbers. Data obtained from the BRENDA data base [17] for about hundred *E. coli *enzymes (Additional file 1) shows that the turnover numbers vary over five orders of magnitude (Fig. 1a), from 10^-2 ^to 10^2 ^1/s. Using these parameter estimates we compute the crowding coefficients *a*_*i *_for about a hundred *E. coli *enzymes (Fig. 1b), resulting in an average and standard deviation of 0.014 and 0.009 1/[mmol/g/h], respectively. Because of the large enzyme turnover variations the crowding coefficients are distributed over a wide range as well, from 10^-6 ^to 10^0 ^1/[mmol/g/h] (Fig. 1b).

### FBAwMC predicts a change of effective metabolic efficiency objective

**Figure 2:**
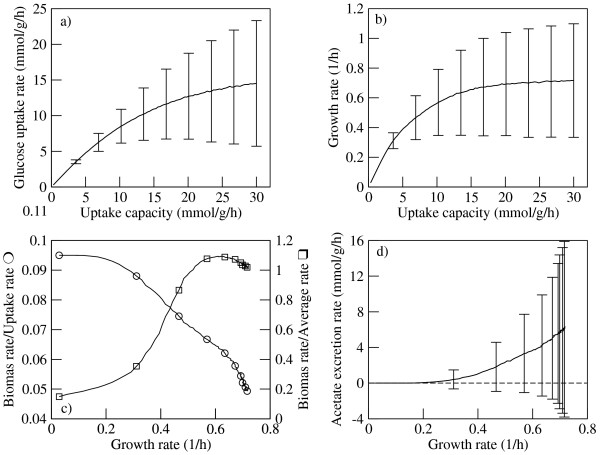
**The signatures of the predicted metabolic switch**: The glucose uptake rate (a) and growth rate (b) as a function of the glucose uptake capacity, as obtained from the FBAwMC model. The line represents the average behavior and the error bars represent the standard deviation over 1,000 choices of crowding coefficients. (c) Flux ratios illustrating the switch in metabolic efficiency objective from low to high growth rates. At low growth the biomass rate per unit of uptake rate (circles) is at a maximum, while the biomass rate per unit of average rate is at a maximum at high growth rates (squares). (d) Acetate excretion rate as a function of the growth rate. At high growth rates the prediction for acetate excretion is sensitive to the crowding coefficients uncertainty, resulting in the large error bars.

Having estimated the crowding coefficients we next evaluate the relevance of the solvent capacity constraint (Eq. 3) at physiological growth conditions. To this end we utilize a FBA model of *E. coli *MG1655 metabolic network that takes into account this constraint referred to as 'flux balance analysis with molecular crowding' (FBAwMC) [12]. Under conditions of aerobic growth in a glucose-limited medium, FBAwMC predicts a saturation of the glucose uptake rate and the growth rate (Fig. 2a,b) with increasing the glucose uptake capacity. The predicted maximum glucose uptake rate (~15 mmol/g/h) and maximum growth rate (~0.7 h^-1^) are within the range of experimentally determined values [18], corroborating our previous report [12] that the solvent capacity constraint (Eq. 3) is relevant at physiological conditions.

Associated with the predicted saturation of *E. coli *metabolic rates, FBAwMC predicts a metabolic switch characterized by a change in the effective criteria of metabolic efficiency. At low growth rates the ratio between the biomass production rate and the glucose uptake rate is at a maximum but decreases with increasing the growth rate. In contrast, the ratio between the biomass production rate and the average reaction rate increases with increasing the growth rate, reaching a maximum at high growth rates. In agreement with our expectations, at low growth rates nutrients are scarce and the best strategy for a cell is to maximize the biomass production rate per unit of limiting nutrient (in this case, glucose) uptake rate. In contrast, at high growth rates the nutrients are abundant, the predicted metabolic rate is limited by the solvent capacity constraint (Eq. 3) and, therefore, the maximum growth rate is achieved by maximizing the biomass production rate per average reaction rate (Fig. 2c). The predicted change in metabolic efficiency objective is accompanied by a redistribution of the metabolic fluxes, including those of exchange fluxes. Indeed, a characteristic example is the predicted excretion of acetate at high growth rates (Fig. 2d) that is well supported by experimental observations [9,19,20].

### FBAwMC-predicted metabolic fluxes are within the range of experimental values

**Figure 3:**
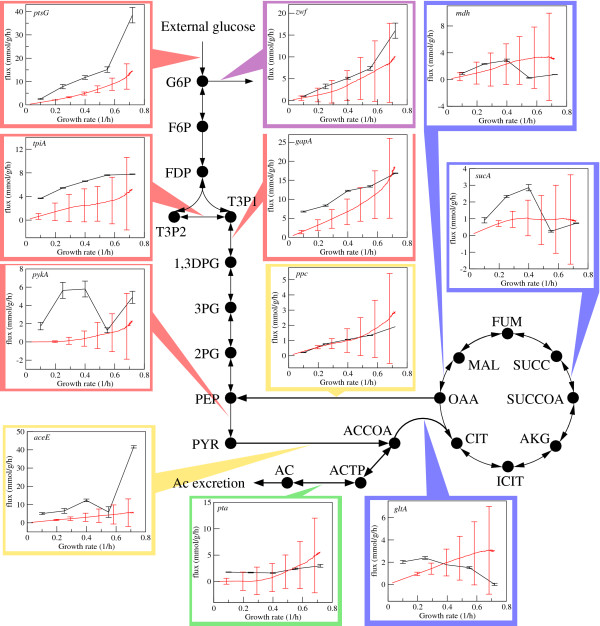
**Predicted vs. measured metabolic fluxes in the *E. coli *central metabolism**. Comparisons between the FBAwMC-predicted- (orange plots) and measured (blue plots) fluxes as a function of growth/dilution rates for selected reactions in the central carbon metabolism of *E. coli*. The experimental flux measurements were performed at dilution rates 0.1, 0.25, 0.4, 0.55 and 0.72 h^-1^. Selected reactions of glycolysis (red boxes), the first reaction of the pentose phosphate pathway (*zwf*) (magenta box), the TCA cycle (blue boxes), acetate excretion pathway (green boxes) and the reactions catalyzed by *ppc *and *aceE *connecting the glycolytic- and TCA pathways (yellow boxes) are shown. The solid black circles represent the denoted metabolites while the black arrows represent metabolic reactions labeled by the genes encoding the enzymes catalyzing the respective reactions (see Table S1 in Additional file 4 for the list of abbreviations and information on enzymes encoded by listed genes). The error bars for predicted fluxes indicate the standard deviation over 1,000 choices of the crowding coefficients among the list of values estimated for ~100 *E. coli *enzymes, whereas the error bars for the experimental fluxes represent the standard deviations for three independent measurements.

FBAwMC is also able to predict internal metabolic fluxes as a function of the growth rate. A subset of the FBAwMC-derived flux predictions in the central carbon metabolism are shown in Figure 3. In most cases the FBAwMC predicted fluxes are within the range of experimentally determined values. This is a striking result given that this implementation of FBAwMC does not contain any free paremeters. The only model parameters are the crowding coefficients, which were determined above using independent experimental results. We should also note that the observed wide variability around the average behavior (Fig. 3, experimental error bars) is not a shortcoming of our modeling framework but is due to our current inability to obtain a direct estimate for all crowding coefficients (due to incomplete information available on various kinetic parameters). Thus, further testing of our predictions will be necessary upon availability of better estimates for the crowding coefficients.

Limiting our analysis to the expected behavior, we observe a slope change for several fluxes when reaching the highest growth rates. The reactions of the glycolytic pathway, the flux towards the pentose-phosphate pathway via the reaction catalyzed by the gene product of *zwf*, and the acetate pathway switch at high growth rates to a faster flux increase with increasing the growth rate (Fig. 3). The experimental values corroborate this qualitative behavior, but the change is bigger for the *ptsG*-catalyzed reaction and even qualitatively different for the *pykA*-catalyzed reaction, both being part of the glycolytic pathway. A second noticeable effect is the predicted saturation of the TCA cycle flux at high growth rates. The experimentally measured values of the TCA cycle flux exhibit, however, a stronger effect characterized by a decreasing tendency at high growth rates (Fig. 3). Taken together these results indicate that while for most reactions the FBAwMC predictions are within the range of experimental measurements, a method for a more accurate estimate of the crowding coefficients on a network scale will be required to provide more precise predictions.

### Identifying the regulatory mechanism(s) that control the action of the metabolic switch

**Figure 4:**
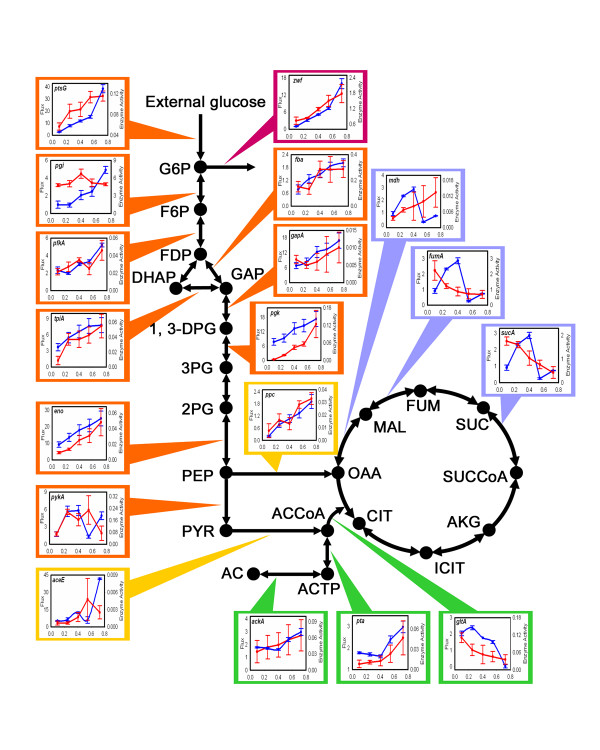
**Comparison of measured metabolic fluxes and *in-vitro *enzyme activities**. Measured flux rates (blue, mmol/h/g dry biomass) and *in vitro *enzyme activities (red, U/mg protein) on two separate Y-axes of selected reactions in the central metabolism of *E. coli *are shown as a function of growth/dilution rates (X-axis). All labels are as in Figure 3. Additional file 2 shows the actual data on measured enzyme activities. The error bars for the experimental flux and enzyme activity plots are a result of three independent measurements.

To examine if the changes in growth conditions and the corresponding adjustments in cellular metabolism can be traced by distinct molecular signatures we next measured the *in vitro *activity of eighteen selected enzymes (Additional file 2) that catalyze reactions in the central carbon metabolism of *E. coli *MG1655, and correlate their changes with those observed for the measured flux rates (Fig. 4). For several enzymes there is a good correlation between the measured enzyme- and flux activities (Pearson Correlation Coefficient, PCC, close to- or larger than 0.8). For example, with an increasing growth rate the enzyme activity of the *ptsG *and *pfkA *gene products follow the same increasing tendency as the fluxes of the corresponding metabolic reactions (PCC = 0.79 and 0.85, respectively). (The glycolytic flux is known to be controlled by the activity of these two enzymes while other reactions adjust their fluxes through changes in metabolite concentrations [21]). In contrast, we found no significant correlation between the measured fluxes and enzyme activities of the TCA reactions (PCC = 0.64, 0.35, -0.03 and -0.28 for enzymes associated with *gltA, sucA*, *fumA *and *mdh*, respectively), implying that the TCA flux is controlled by the activity of enzymes catalyzing reactions outside this pathway. A possible candidate to exert this action is the acetate pathway. Indeed, an increase of the flux on the acetate pathway towards the production of acetate can balance both the increase in the flux originating from the glycolytic pathway through *aceE *and a decrease in the flux from Acetyl-CoA to the TCA cycle. This hypothesis is supported by the increase in the enzyme activity of phosphotransacetylase (*pta*) when the growth rate increases beyond 0.4 h^-1 ^(PCC = 0.98), which is exactly the growth rate threshold where the switch is taking place.

In parallel with the enzyme activity measurements we also prepared mRNA from samples obtained at all five dilution rates and processed them for microarray analysis. The full microarray data set is presented in the Additional file 3 and its detailed analysis is presented in Additional file 4 (SI text 5–8). In contrast to the observed overall correlation between measured fluxes and *in vitro *enzyme activities we do not observe a significant correlation between the measured metabolic fluxes and the relative changes in mRNA levels of enzyme-encoding genes (SI Fig. S5 in Additional file 4), implying that the switch and corresponding enzymatic functions are not predominantly controlled at the transcriptional level. Correspondingly, no significant correlation between the *in vitro *enzyme activities and the relative changes in mRNA levels of enzyme-encoding genes can be seen (SI Fig. S5 in Additional file 4). Taken together these results indicate that the metabolic switch is predominantly controlled by an increase in the enzyme activities of the end products of *ptsG *and *pfkA *controlling the glycolysis flux, and *pta *controlling the acetate pathway flux, respectively.

## Discussion

Developing a modeling framework that can describe and predict in a quantitative manner the experimentally observed behavior of an organism is a significant challenge for systems biology. One prerequisite of this goal is to uncover the physicochemical constraints exerting the main influences on cellular metabolism [4]. Our results here and in Ref. [12] indicate that the limited solvent capacity represents a physiologically relevant constraint for fast growing *E. coli *cells. The incorporation of this constraint to the FBA modeling framework leads to the FBAwMC model whose predictions indicate that the solvent capacity constraint results in a maximum glucose uptake rate and growth rate that are within the range of experimentally determined values. The flux predictions for several reactions of the *E. coli *metabolism are within the range of our measurements, as well.

From the perspective of quantitative modeling using flux balance approximations, the solvent capacity constraint forces us to consider reaction kinetics via the crowding coefficients, at least for fast growing cells. At low metabolic rates the solvent capacity constraint is less relevant and flux balance alone is sufficient to obtain satisfactory predictions. In contrast, at high metabolic rates a precise knowledge of the crowding coefficients is required to obtain accurate predictions. In the absence of kinetic information we can still obtain a good approximation by sampling the crowding coefficients from a list of estimated values and then focus on the resulting general trend.

More importantly, the solvent capacity constraint allows the interpretation of the metabolic switch taking place between slow and fast growing *E. coli *cells. A recent study of FBA models with different objectives demonstrates that under nutrient scarcity a FBA model with the maximization of the biomass yield objective achieve the highest predictive accuracy, while maximizing the ATP or biomass yield per average flux unit is the best objective for unlimited growth on glucose under aerobic conditions [22]. In contrast, by considering the solvent capacity constraint we obtain the same results using the maximization of biomass production rate objective alone (Fig. 2c). This is more consistent with the expectation that cells achieving the fastest growth rates outgrow cells growing at a slower rate, but how the highest growth rate is achieved is determined by both the availability of substrates and internal metabolic constraints, such as the solvent capacity. Furthermore, the well-known acetate excretion [9,19,20] is explained by the solvent capacity constraint as well. We should note, however, that this does not exclude the possibility that under certain physiological conditions acetate excretion may result from a limited availability of oxygen in the culture medium [23].

## Conclusion

From a more general perspective the results reported in this study further implicate the limited solvent capacity as an important constraint acting on the metabolism of cells operating at high metabolic rates. Thus, the relevance of this constraint will also need to be examined in the context of the observed aerobic ethanol excretion of fast growing yeast cells (Crabtree effect) [24] and aerobic glycolysis in fast growing mammalian cells, particularly tumor cells (Warburg effect) [25]. The common theme of these effects is that (i) they occur in fast growing cells and (ii) that in the presence of oxygen cells partially switch to anaerobic metabolism, resulting in the excretion of metabolic byproducts. Yet, the role of other proposed effects, such as a limit on attainable mitochondrial respiration [26] and available oxygen [23], cannot be excluded and, therefore, should be subject to further studies.

## Methods

### Estimation of crowding coefficients

The *E. coli *intracellular density is *C *= 0.34 g/ml [15]. The specific volume was estimated for several proteins using the molar volumes and masses reported in Ref. [16], resulting in average of 0.73 ml/g and standard deviation of 0.02 ml/g. The enzymes' turnover rates were obtained from the BRENDA database [17] for 102 *E. coli *enzymes.

### Metabolic flux predictions

The Flux Balance analysis with Molecular Crowding [12] is implemented by solving the following optimization problem: maximize the biomass production rate subject to the constraints: balance in the production and consumption of each metabolite (flux balance), the maximum capacity constraint for the carbon source uptake rate and the solvent capacity constraint (Eq. 3). After expressing the reaction's stoichiometric coefficients in units of mol/dry biomass, the maximum growth rate corresponds to the biomass production rate, where biomass production is an auxiliary reaction containing as substrates the cellular components in their relative concentrations and as product the cell's biomass. The crowding coefficients were modeled as noise, assigning them randomly selected values from a list of estimated values for about hundred *E. coli *enzymes (Additional file 1). The predictions for all fluxes are provided in Additional file 5.

### Bacterial strain and general growth conditions

The *E. coli *K12 strain MG1655 (F^- ^λ^- ^*ilv*G *rfb*50 *rph*1) was used throughout the work. In order to obtain biomass samples for flux measurements, 20-ml of the overnight grown culture (~8–10 h) of wild-type cells in LB-medium was inoculated in 980-ml M9 minimal medium (Sigma) containing 2 g/L glucose, where 90% was natural glucose and the remaining 10% was labeled glucose [1,2-^13^C_2_]-glucose (with >99% purity and 99% isotope enrichment for each position, [Cambridge Isotope Laboratories, Andover, MA]). Cells were grown in a continuous growth mode at 5 different dilution rates (0.1, 0.25, 0.4, 0.55, and 0.72 L h^-1^) in a Labfors bioreactor (Infors, Switzerland). The growth of the bacterial culture was regularly monitored at A_600nm _to document steady state, as described in Additional file 4 (SI text 3 and SI Fig. S7)

### Metabolic enzyme activity assays

For determining metabolic enzyme activities from three separate experiments, the cell pellets (collected at all five dilution rates) were first resuspended and washed in 100 mM Tris-HCl (pH 7.0) sonication buffer containing 20 mM KCl, 5 mM MnSO_4_, 2 mM DTT and 0.1 mM EDTA, and then disrupted in a sonicator by 3 sonication cycles of 30 sec each. The cell debris was removed by centrifugation and the resulting cell extract (supernatant) was immediately used for enzyme assays or stored at -20°C. All subsequent steps were carried out on ice. Supernatants of the samples were used for quantitative assaying of endogenous enzyme activities by continuous spectrophotometric rate determination at 30°C in a thermostatically controlled spectrophotometer (Cary 500) with 1-cm light path, as described in the Additional file 4 (SI text 3). Supernatants of the samples were also used for determining total protein concentrations using standard Bradford's assay (BioRad).

### Flux measurements and analyses

For flux analysis, biomass (from ~100 ml culture) and supernatant samples were collected at all five dilution rates. These samples were immediately flash frozen in liquid nitrogen and stored at -80°C until further analysis. Flux rates were determined using a tracer-substrate based GC-MS and NMR metabolome mapping platform. The analyses included determining positional ^13^C tracer enrichment in multiple intermediary metabolites of glycolysis, glycogen synthesis, tricarboxylic acid cycle and their intracellular products from [1,2-^13^C_2_]-D-glucose, as described in detail in Additional file 4 (SI text 4). The retention times and mass-to-charge (m/z) ion clusters of selected ions of bacterial and culture media metabolites were determined using mass isotopomer analysis (MIDA) [27,28], and expressed as net fluxes by subtracting reverse fluxes from forward tracer incorporation patterns via reversible metabolic steps [29]. Results were expressed as mmol/hr/g dry biomass glucose. Each experiment was carried out using triplicate cell cultures for each condition within each experiment, and the experiments were repeated once. Mass spectroscopic analyses were carried out by three independent automatic injections of 1 μl samples by the automatic sampler and accepted only if the standard sample deviation was less than 1% of the normalized peak intensity. Statistical analysis was performed using the Student's t-test for unpaired samples. Two-tailed significance at the 99% confidence interval (μ +/- 2.58σ), p < 0.01 indicated significant differences in glucose-derived fluxes. For some reversible reactions, we measured both forward and reverse fluxes and calculated net fluxes towards product synthesis.

### RNA preparation for microarray analysis

At all five dilution rates, 10–20 ml of the cell culture was collected, mixed with 10% (v/v) of ice cold stop-solution (5% water-saturated phenol in absolute ethanol), and cell pellets were obtained by centrifugation at 4,500 × *g *for 5 min at 4°C, followed by flash-freezing of pellets with liquid nitrogen. Cell pellets were stored at -80°C until further use. RNA was isolated from the frozen cell pellets using Masterpure RNA isolation kit (Epicentre Biotechnologies, Madison, WI) and RNA samples were processed for transcriptome analysis using *E. coli *Affymetrix microarray chips, as described previously [12]. Dchip method was used to analyze all microarray data as described in Additional file 4 (SI text 5–8).

## Authors' contributions

AV, MAM, and A-LB developed the theoretical framework and AV implemented it. QKB and LGB performed the experiments, with the exception of the microarray experiments that were performed at the Boston University Microarray Center. JE and ZB-J analyzed the microarray data. AV and ZNO guided and coordinated the project. AV, QKB, ZB-J, LGB, and ZNO prepared the manuscript. All authors have read and approved the final version of the manuscript.

## Supplementary Material

Additional file 1 *E. coli* metabolic reactions and biomass vector Click here for file

Additional file 2 Measured enzyme activity values for various intracellular enzymes of the central carbon metabolism Click here for file

Additional file 3 Dchip Microarray analysis of biomass samples from all five dilution/growth rates Click here for file

Additional file 4 Impact of the solvent capacity constraint on *E. coli *metabolism Click here for file

Additional file 5 Flux predictions for 710 reactions in the *in silico *of *E. coli *metabolism during growth on glucose-limited medium Click here for file

Additional file 6 List of all genes along with their 'b'-numbers showing similar expression profile with query genes. Click here for file
