# Supplementary material for: Impact of the solvent capacity constraint on E. coli metabolism
Source: BMC Syst Biol. 2008 Jan 23;2:7. doi: 10.1186/1752-0509-2-7 (PMC2270259; doi:10.1186/1752-0509-2-7)
Supplement: Additional file 4 — Impact of the solvent capacity constraint on E. coli metabolism [file 1752-0509-2-7-S4.DOC]

# Impact of the solvent capacity constraint on *E. coli* metabolism

# Detailed Supporting Information

Alexei Vazquez^1*^, Qasim K. Beg^2*+^, Marcio A. deMenezes^3^, Jason Ernst^4^, Ziv Bar-Joseph^4^, Albert-László Barabási^5^, László G. Boros^6^ and Zoltán N. Oltvai^2^

^1^The Simons Center for Systems Biology, Institute for Advanced Study, Princeton, NJ 08540, USA

^2^Department of Pathology, University of Pittsburgh, Pittsburgh, PA, 15261, USA

^3^Instituto de Física, Universidade Federal Fluminense, Rio de Janeiro*,* 24210, Brazil

^4^ SiDMAP, LLC and the UCLA School of Medicine, Los Angeles, CA 90064, USA

^5^Machine Learning Department, Carnegie-Mellon University, Pittsburgh, PA, 15217, USA

^6^Department of Physics and Center for Complex Networks Research, University of Notre Dame, South Bend, IN 46556, USA

* these authors contributed equally to this work

+ current address: Department of Biomedical Engineering, Boston University, Boston, MA 02215

Correspondence and requests for materials should be addressed to A.V. ([vazquez@ias.edu](mailto:vazquez@ias.edu)) or Z.N.O. ([oltvai@pitt.edu](mailto:oltvai@pitt.edu)).

**SI Text 1: Theoretical description of flux predictions in other substrates**

Following the same procedure as for glucose (Additional file 5), we also made predictions for the *E. coli* metabolic fluxes when growing on glycerol, lactate and succinate. The increase of the carbon source concentration in the growth medium was modeled as an increase of the maximum capacity of the corresponding carbon source uptake flux. For each maximum capacity we computed the fluxes that maximize the biomass production rate, obtaining a prediction for the optimal flux of all reactions and the optimal biomass production rate. Because the biomass production rate equals the growth rate, using these predictions we can analyze the behavior of metabolic fluxes as a function of the growth rate. This is shown in SI Fig. 6 for selected reactions in the *E. coli* central metabolism.

## SI Text 2. Bacterial strain and general growth conditions

The *E. coli* K12 strain MG1655 (F^-^ *λ*^-^ *ilv*G *rfb*50 *rph*1) was used throughout the work. In order to obtain biomass samples for flux measurements, 20-ml of the overnight grown culture (~8-10 h) of wild-type cells in LB-medium was inoculated in 980-ml M9 minimal medium (Sigma) containing 2 g/L glucose, where 10% was labeled glucose **[1,2-^13^C_2_]-glucose** and the remaining 90% was natural glucose. Cells were grown in a continuous growth mode at 5 different dilution rates (0.1, 0.25, 0.4, 0.55, and 0.72 L h^-1^) over a period of approximately 5 days in a bioreactor (Labfors, Infors AG, Switzerland) (SI Fig. 7). The growth of the bacterium was regularly monitored at A_600nm_ to document steady state at all dilution rates. The dissolved oxygen was set at 100% initial value, and sterile air was continuously sparged into the medium. Growth parameters, such as pO_2_, pH, temperature (37°C) and agitation (~400 rpm) were continuously monitored using microprocessor probes. The pH of the medium was constant around 7.0 and was controlled with regular adjustments by automatic supply of acid (10% H_3_PO_4_) and base (2N NaOH) using two peristaltic pumps. For determining intracellular metabolic enzyme activities and global transcriptome profiles, the bacterium was grown under similar conditions (except only natural glucose was used as source of carbon) in three separate experiments, and biomass samples were collected at all five-dilution rates. Biomass samples for intracellular enzyme activity, gene expression and flux determination were harvested at the end of each major dilution rates indicated by constant A_600nm_ (optical densities) and pO_2_ concentrations in the growth medium (SI Fig. 7).

## SI Text 3. Enzyme activity assays

The 30-ml samples for enzyme assays were collected for various dilution rates at the end of each dilution rate (indicated by different colors in SI Fig. 7). The cells pellets for enzyme assays were harvested by centrifugation at 4,000 *x g* at 4°C for 10 min. These cell pellets were re-suspended and washed in 100 mM Tris-HCl (pH 7.0) sonication buffer (Peng and Shimizu 2003) containing 20 mM KCl, 5 mM MnSO_4_, 2 mM DTT and 0.1 mM EDTA. The cells were disrupted by 3 sonication cycles of 30 sec each in a sonicator (Fisher Scientific) to recover maximum possible yield of enzyme. The cell debris was removed by centrifugation and the resulting cell extract (supernatant) was immediately used for enzyme assays or stored at -20°C. All operations were carried out on ice. The supernatant of this sample was used to determine total protein concentration in enzyme samples using standard Bradford’s assay (Biorad, Richmond, CA). This sample was also used for estimation of quantitative assay of endogenous enzymes. The method of continuous spectrophotometric rate determination with time was followed for measurement of activities of the enzymes of metabolic pathways. All the enzyme assays were done at 30°C in a thermostatically controlled UV/Vis spectrophotometer (Cary 500) with 1-cm light path. All components the reaction mixture and respective substrates were pipetted out in a quartz cuvette (Fisher Scientific) and blanks were adjusted. Reactions were initiated by adding supernatant from the sonicated enzyme samples to give a final volume of 1 ml. The millimolar extinction coefficients for NAD^+^, NADH, NADP^+^ and NADPH was 6.22 cm^-1^ mM^–1^ at 340 nm, and those of methyl viologen and benzyl viologen at 578 nm was 9.78 and 8.65 cm^-1^.mM^-1^, respectively, whereas the millimolar extinction coefficient value for 5-mercapto-2-nitrobenzoic acid at 412 nm was 13.6 cm^-1^ mM^-1^. For all enzyme assays described below, we define 1 unit of enzyme as the amount of enzyme required to convert 1 μmol of substrate into specific products per minute per milligram of protein under defined conditions of pH and temperature. Most enzyme assay protocols used were the standard assay protocols from Sigma (St. Loius, MO, USA), expect few, which were obtained from the published literature (Peng and Shimizu 2003; Van der Werf et al. 1997; Zhao et al. 2004).

The assay conditions for various enzymes were as follows (the gene names are listed in parenthesis against the enzyme name): *Glucose:PEP phosphotransferase (pts)*: 0.1 M Tris-HCl (pH 8.4), 10 mM MgCl_2_, 1 mM DTT, 1 mM NADP^+^, 10 mM d-glucose, 3 U glucose-6-phosphate dehydrogenase, 10 mM PEP. *Phosphofructose kinase (pfkA)*: 50 mM imidazol-HCl (pH 7.0), 0.05 mM ATP, 5 mM MgCl_2_, 1 mM EDTA, 0.25 mM NADH, 0.25 mM fructose-6-phosphate (F6P), 0.5 U aldolase, 0.5 U glyceraldehyde phosphate dehydrogenase, 0.5 U triose phosphateisomerase. *Fructose-1,6-bisphosphate aldolase (fbaA)*: 0.05 M Tris-HCl (pH 7.5), 0.1 mM cysteine-HCl, 0.1 M potassium acetate, 2 mM FDP, 0.7 mM CoCl_2_, 0.25 mM NADH, 20 U triose phosphate isomerase, 2 U glycerol-3-phophate dehydrogenase. *Glyceraldehyde-3-phosphate dehydrogenase (gapA):* 0.1 mM Tricine-HCl (pH 8.1), 5 mM potassium phosphate, 20 mM neutralized sodium arsenate, 2 mM FDP, 2 mM DTT, 1 mM NAD^+^, 1 U aldolase. *Triosephosphate isomerase (tpiA):* 300 mM triethanolamine buffer (pH 7.8), 0.2 mM NADH, 1 U glycerolphosphate dehydrogenase, 5 mM glyceraldehyde-3-phosphate. *Phosphoglycerate kinase (pgk):* 0.1 M triethanolamine buffer (pH 7.8), 1 mM EDTA, 2 mM MgSO_4_·7H_2_O, 1 U glyceraldehyde-phosphate dehydrogenase, 1 mM ATP, 10 mM 3-phosphoglycerate. *Pyruvate kinase (pykA):* 0.1 M Tris-HCl (pH 7.5), 5 mM ADP, 1 mM DTT, 10 mM KCl, 15 mM MgCl_2_, 0.5 mM phosphoenol pyruvate, 0.25 mM NADH, 10 U lactate dehydrogenase. *α-Ketoglutarate dehydrogenase (sucA):* 0.2 M phosphate buffer (pH 7.2), 1 mM CoASH, 0.1 M cysteine-HCl (pH 7.2), 10 mM NAD^+^ (pH 7.2), 3 mM α-ketoglutarate. *Fumarase (fumA):* (assay based on formation of fumarate at 240 nm) 0.1 M Tris-HCl buffer (pH 7.2), 50 mM l-malate. *Malate dehydrogenase (mdh):* 2.5 ml 0.1 M Tris-HCl (pH 8.8), 0.1 ml 0.1 mM sodium malate, 0.1 ml 10 mM NAD^+^ and cell extract, and water to a final volume of 3 ml. *Phosphotransacetylase (pta):* 0.1 M Tris-HCl (pH 7.8), 0.2 mM CoA, 30 mM NH_4_Cl, 1.0 mM DTT, 1.0 mM NAD^+^, 5.0 mM l-malate, 4 U citrate synthase, 20 U malate dehydrogenase, 2.0 mM acetyl phosphate. *Acetate kinase (ackA):* 0.1 M Tris-HCl (pH 7.4), 0.8 M potassium acetate, 0.2 M KCl, 4 mM ATP, 4 mM MgCl_2_, 1.6 mM PEP, 0.4 mM NADH, 4 U pyruvate kinase, 55 U lactate dehydrogenase; *Citrate synthase (gltA):* 100 mM Tris-HCl (pH 8.0), 8 mM Acetyl CoA, 10 mM sodium oxaloacetate, 10 mM 5,5’-dithiobios-2-notrobenzoate; *Glucose-6-phosphate dehydrogenase (zwf):* 100 mM Tris-HCl (pH 7.5), 2.5 mM MnCl_2_, 2 mM glucose-6-phosphate, 1 mM DTT, 1 mM NADP^+^; *PEP carboxylase (ppc):* 66 mM Tris-HCl (pH 9.0), 10 mM MgCl_2_, 10 mM sodium bicarbonate, 0.15 mM NADH, 2 U malate dehydrogenase, 5 mM phosphoenolpyruvate; *Pyruvate dehydrogenase (aceE):* 100 mM Tris-acetate (pH 7.8), 5 mM pyruvate, 0.1 mM CoA, 7 mM sodium arsenate, 2 mM methyl viologen; *Enolase (eno):* 100 mM Triethalomine buffer (pH 7.4), 5.6 mM phosphoglycerate, 0.35 mM β-NADH, 75 mM MgSO_4_, 300 mM KCl, 1 mM ADP, 0.1 ml solution of 15 mM Tris-HCl (pH 7.4) mixed with 0.2% BSA. *Phosphoglucose isomerase (pgi):*  100 mM Tris-HCl (pH 7.8), 10 mM MgCl_2_, 0.5 mM NADP^+^, 10 U glucose-6-phosphate dehydrogenase, 2 mM fructose-6-phosphate.

The following equation was used for calculating enzyme activities for most of the enzyme (unless specified)

where, *V_a_*: total volume (ml) of assay; DF: Dilution factor; E.C.: millimolar extinction coefficient of NAD^+^, NADH, NADP^+^ or NADPH at 340nm was 6.22 cm^-1^.mM^-1^, and those of methyl viologen and benzyl viologen at 578 nm was 9.78 and 8.65 cm^-1^.mM^-1^, respectively, whereas it was 13.6 cm^-1^.mM^-1^ for 5-mercapto-2-nitrobenzoic acid at 412 nm. *V_e_:* Volume of enzyme

ΔA_340nm_/min (Test) - ΔA_340nm_/min (Blank)

Units/ml of enzyme =

E.C. x *V_e_*

x *V_a_* x DF

Units/ml enzyme

Units/mg protein =

mg protein/ml

**SI Text 4: Intracellular flux measurements**

*Glycogen glucose and RNA ribose stable isotope studies:* RNA ribose and glycogen glucose were isolated by acid hydrolysis of cellular RNA after Trizol purification of cell extracts. Total RNA amounts were assessed by spectrophotometric determination, in triplicate cultures. Ribose and glycogen glucose were derivatized to their aldonitrile acetate form using hydroxylamine in pyridine with acetic anhydride (Supelco, Bellefonte, PA) before mass spectral analyses. We monitored the ion cluster around the m/z256 (carbons 1-5 of ribose) (chemical ionization, CI) and m/z217 (carbons 3-5 of ribose) and m/z242 (carbons 1-4 of ribose) (electron impact ionization, EI) to determine molar enrichment and the positional distribution of ^13^C in ribose. For glycogen glucose we monitored m/z327-332 using CI. By convention, the base mass of ^12^C- compounds (with their deriviatization agents) is given as m_0_ as measured by mass spectrometry as described elsewhere (Boros et al. 2002). Ribose or glucose molecules labeled with a single ^13^C atom on the first carbon position (m1) recovered from RNA or glycogen, respectively, were used to gauge the ribose fraction produced by direct oxidation of glucose through the G6PD pathway. Ribose molecules labeled with ^13^ C on the first two carbon positions (m2) were used to measure the fraction produced by transketolase. Doubly labeled ribose molecules (m_2_ and m_4_) on the fourth and fifth carbon positions were used to measure molar fraction produced by triose phosphate isomerase and transketolase.

###### *Lactate:* Lactate from the cell culture media (0.2 ml) was extracted by ethylene chloride after acidification with HCL. Lactate was derivatized to its propylaminehepta- fluorobutyrate ester form and the *m/z328* (carbons 1-3 of lactate) (chemical ionization, CI) was monitored for the detection of *m_1_* (recycled lactate through the PC) and *m_2_* (lactate produced by the Embden-Meyerhof-Parnas pathway) for the estimation of pentose cycle activity (Lee et al. 1998a). In this study we recorded the *m_1_*/*m_2_* ratios in lactate produced and released by bacterial cells in order to determine pentose cycle activity versus anaerobic glycolysis.

###### *Glutamate:* Glutamate label distribution from glucose is suitable for determining glucose oxidation versus anabolic glucose use within the TCA cycle, also known as anaplerotic flux. Tissue culture medium was first treated with 6% perchloric acid and the supernatant was passed through a 3 cm^3^ Dowex-50 (H+) column. Amino acids were eluted with 15 ml 2N ammonium hydroxide. To further separate glutamate from glutamine, the amino acid mixture was passed through a 3 cm^3^ Dowex-1 (acetate) column, and then collected with 15 ml 0.5 N acetic acid. The glutamate fraction from the culture medium was converted to its trifluoroacetyl butyl ester (TAB). Under EI conditions, ionization of TAB-glutamate produces two fragments, *m/z198* and *m/z152,* corresponding to C2-C5 and C2-C4 of glutamate (Lee et al. 1996). Glutamate labeled on the 4-5 carbon positions indicates pyruvate dehydrogenase activity while glutamate labeled on the 2-3 carbon positions indicates pyruvate carboxylase activity for the entry of glucose carbons to the TCA cycle. TCA cycle anabolic glucose utilization is calculated based on the *m_1_/m_2_* ratios of glutamate (Leimer et al. 1977).

*Fatty acids:* Palmitate, stearate, cholesterol and oleate were extracted after saponification of cell pellets in 30% KOH and 100% ethanol using petroleum ether. Fatty acids were converted to their methylated derivative using 0.5N methanolic-HCL. Palmitate, stearate and oleate were monitored at *m/z270, m/z 298* and *m/z264*, respectively, with the enrichment of ^13^C labeled acetyl units which reflect synthesis, elongation and desaturation of the new lipid fraction as determined by mass isotopomer distribution analysis (MIDA) of different isotopomers (Lee et al 1998b; Lee et al 1995).

*Gas Chromatography/Mass Spectrometry (GC/MS):* Mass spectral data were obtained on the HP5973 mass selective detector connected to an HP6890 gas chromatograph. The settings were as follows: GC inlet 250^o^C, transfer line 280^o^C, MS source 230^o^C, MS Quad 150^o^C. An HP-5 capillary column (30m length, 250μm diameter, 0.25μm film thickness) was used for glucose, ribose and lactate analyses.

*^13^C, ^1^H and ^31^P Nuclear Magnetic Resonance studies of intracellular metabolites:* Nuclear Magnetic Resonance (NMR) studies included acetate, alanine, betaine, cholines creatine, glucose, glutamate, glutamine, total glutathion (GSH), glycine, 3-hydroxybutyrate, myo-inositol, lactate, phosphocreatine, pyruvate, valine, hosphocreatine, creatine, ATP, ADP, AMP, NAD+, and total phosphomonoesters (PME) and phosphodiesters (PDE) extracted by ice-cold 0.9% NaCl, 12 % PCA and 8 M KOH. The procedure included the transferring of 5 mL medium into a 15-mL tube (ice-bath), removing the rest of the medium thoroughly, washing cells with 6 mL of ice-cold NaCl, adding 2 mL ice-cold 12% PCA to the frozen cells or media and spinning at 1300 g for 20 min at 4°C. After transferring the supernatant into a new 50 ml tube and resuspending the pellet in 2 mL ice-cold 12% PCA in the old 50 mL tube we placed the tubes in an ultrasound ice bath for 5 min. We collected the supernatants, and lyophilized them in a freeze-dry system overnight. After re-suspending ^13^C, ^1^H and ^31^P spectra were obtained on a 9T Brucker vertical bore instrument for quantitative and ^13^C positional analyses (Gottschalk et al. 2004; Serkova et al. 2003, 2005).

*Tricarboxylic acid cycle analysis using trimethylsilyl (TMS) derivatives:* Frozen pellets (-80 **^O^**C; wet 0.5 g) were powderized and extracted with 2:1 (volume) of chloroform-methanol using Omni-TH homogenizer. The slurry was centrifuged at 670 g for 20 min and the upper methanol-water phase was collected and treated with 200 μl of methoxylamine-HCl to protect keto and aldehyde groups. The lower chloroform phase is vortexed for 5 min with 10 mL of methanol-water 3:2 by volume. After 20 min centrifugation the two upper methanol-water phases were combined. The combined methanol-water phase was adjusted to pH 8.0 and evaporated under constant flow of Nitrogen gas in an exhaust fume hood. The residue was reacted with 100 μL of bis(trimethylsilyl) trifluoroacetamide with 10% trimethylchlorosilane (Regisil) at 70 **^O^**C for 70 minutes to form the TMS and MOX-TMS derivatives of TCA cycle metabolites. GC-MS analyses were carried out on an Agilent 5975 mass spectrometer, equipped with a model 6890 gas chromatograph and a Varian VF-5MS capillary column (60 m, 0.25 mm i.d., 0.25 mm film thickness (Yang et al. 2006).

*Flux data analysis and statistical methods:* Each experiment was carried out using triplicate cell cultures for each condition within each experiment and experiments were repeated once. Mass spectroscopic analyses were carried out by three independent automatic injections of 1µl samples by the automatic sampler and accepted only if the standard sample deviation was less than 1% of the normalized peak intensity. Statistical analysis was performed using the Student’s t-test for unpaired samples. Two-tailed significance at the 99% confidence interval (μ+/-2.58σ), p<0.01 indicated significant differences in glucose-derived fluxes. For some reversible reactions, we measured both forward and reverse fluxes and calculated net fluxes.

**SI Text 5: Microarray sample collection and analysis at different dilution rates**

The culture samples for microarray analysis were collected at all five dilution rates. Approximately, 10 ml of the cell culture was obtained and rapidly mixed with 1/10^th^ volume of the ice-cold stop-solution (5% water-saturated phenol in absolute ethanol) to inhibit any further transcriptional activity. The tubes were capped, and the sample and stop solution were mixed by inversion. The cell pellets were obtained by centrifugation at 4,500 *x g* for 5 min at 4°C, were immediately flash frozen in liquid nitrogen, and were stored at -80°C until further use. RNA was isolated from the frozen cell pellets using Epicenter’s Masterpure RNA isolation kit (using manufacturer’s product manual). The samples were also treated with DNAse for 1 hr at 37°C to remove any DNA contamination in the RNA samples. 10μg of all RNA samples were processed for transcriptome analysis using *E. coli* Affymetrix microarray chips by the Microarray Resource Centre, Department of Genetics and Genomics at Boston University School of Medicine as described previously (Beg et al. 2007). The microarray data was normalized using dChip (Li and Wong 2001). The detailed microarray data from five dilution rates is presented in the Additional file 3.

**SI Text 6: STEM Clustering Analysis**

We used the Short Time-series Expression Miner (STEM) (Ernst and Bar-Joseph, 2006) to identify significant patterns in gene expression profiles in response to the increasing dilution rates. The gene expression profiles were transformed so that they represent the log ratio change in expression from the first sample. The STEM clustering method identifies from a comprehensive library of distinct profiles those with a statistically significant number of genes most closely matching the shape of the profile. Significant profiles are then grouped together such that all profiles in the same group are similar within a threshold. The clusters of significant profiles are then analyzed with a Gene Ontology (GO) enrichment analysis. SI Fig. 8 shows the library of profiles considered and the significant profiles at a 0.05 Bonferroni corrected level in color. Significant profiles that were grouped together are in the same color.

In SI Figs. 9-13, we show genes from significant profiles organized by their clustering group. We also show a GO enrichment table for the set of genes assigned to each of the clusters. The GO categories assigned to various clusters in SI Figs. 9-13 reveals important results with respect to the changing gene expression profile at the five dilution rates. We found that the expression profiles for most of the genes in Cluster 1 (SI Fig. 9) are very similar to the expression profiles for the genes related to the TCA cycle (Figure 5 main manuscript). For this cluster of genes we see that the GO categories cellular biosynthetic and metabolic processes have significant p-values. In Cluster 2 (SI Fig. 10) we see that genes for other cellular processes such as flagella, fimbrium development, cell adhesion, and activities in the outermembrane-bound periplasmic are down-regulated at all dilution rates until 0.55 h^-1^; and the genes for most of these processes were up-regulated at the highest measured dilution rate of 0.72 h^-1^. Analysis of Cluster 3 (SI Fig. 11) reveals that activities of most of the genes responsible for part of cell membrane and porin activities are up regulated at intermediate dilution rates of 0.4 h^-1^ followed by a down regulation at 0.55 h^-1^ and up-regulation again at 0.72 h^-1^. In Cluster 4 (SI Fig. 12) we see that the genes responsible for synthesis and metabolism of various kinds of polysaccharides, biopolymers and lipids, and DNA-mediated transposition were always down-regulated during the course of intermediate growth rates, except at the highest growth rates (0.72 h^-1^), which means that *E. coli* never had the requirement of synthesizing or metabolizing polysaccharides at intermediate growth rates. Analysis of Cluster 5 (SI Fig. 13) reveals that the genes responsible for biosynthesis and metabolism of various amino acids, (such as glutamine, histidine, arginine etc.), urea cycle, and metabolism of various carboxylic acids are always up regulated throughout the growth of the bacterium at all dilution rates.

**SI Text 7. Querying expression data to identify specific expression profiles**

To assess the quality of the microarray profiles and to identify genes with expression patterns that are similar to genes encoding enzymes of the central carbon metabolism, we used TimeSearcher (Hochheiser et al. 2003) to identify genes having similar expres­sion profiles to any of the 23 genes corresponding to the 18 enzymes of Figure 4 (main manuscript). TimeSearcher displays a set of genes satisfying constraints imposed by visual query boxes. The input is a set of known genes and a set of constraint boxes around these genes, and the output is these genes plus all the other genes that also satisfy the constraint boxes. In SI Figs. 14-36, the query boxes are the blue boxes and a known (query) gene corresponding to the enzyme is displayed in green. The list of genes with similar expression pattern identified for each profile (see SI Figs. 14-36) are listed in Additional file 6.

**SI Text 8: Querying gene expression of operons in the central carbon metabolism**

We also examined the expression profile obtained at five-dilution rates growth of *E. coli* organized in the operons in the central carbon metabolism. Apart from the genes already listed in SI Fig. 5, and in SI Figs. 37-41, we show additional genes that are organized in operons (eg. *frdABCD, sdhABCD, aceABK*) for several more reactions in the central carbon metabolism, for which we don't have measured flux value. These plots indicate a good agreement in the expression pattern of genes from the same operon.

**Supplementary Table S1:** List of abbreviations used in the manuscripts and Figs. 3,4 and S5

| **Substrates** | **Abbreviations** |
| --- | --- |
| **G6P** | GLUCOSE-6-PHOSPHATE |
| **F6P** | FRUCTOSE-6-PHOSPHATE |
| **FDP** | FRUCTOSE-1,6-DIPHOSPHATE |
| **DHAP** | DIHYDROXY ACETONE PHOAPHATE |
| **GAP** | HLYCERALDEHYDE PHOSPHATE |
| **1,3-DPG** | 1,3-DIPHOSPHO GLYCERATE |
| **3-PG** | 3-PHOSPHOGLYCERATE |
| **2-PG** | 2-PHOSPHOGLYCERATE |
| **PEP** | PHOSPHOENOL PYRUVATE |
| **PYR** | PYRUVATE |
| **AC** | ACETATE |
| **ACCOA** | ACETYL COENZYME A |
| **ACTP** | ACETYL PHOSPHATE |
| **OAA** | OXALOACETATE |
| **CIT** | CITRATE |
| **ICIT** | ISOCITRATE |
| **AKG** | ALPHA-KETOGLUTARATE |
| **SUCCoA** | SUCCINYL COENZYME A |
| **SUC** | SUCCINATE |
| **FUM** | FUMARATE |
| **MAL** | MALATE |
| **Genes** | **Encoded enzyme** |
| ***ptsG*** | Glucose:PEP phosphotransferase |
| ***pfk*** | Phosphofructokinase |
| ***gapA*** | Glyceraldehyde-3-phosphate dehydrogenase |
| ***tpiA*** | Triosephosphate isomerase |
| ***pgk*** | Phosphoglycerate kinase |
| ***pykA*** | Pyruvate kinase |
| ***sucA*** | a-Ketoglutarate dehydrogenase |
| ***fumA*** | Fumarase |
| ***mdh*** | Malate dehydrogenase |
| ***pta*** | Phosphotransacetylase |
| ***ackA*** | Acetate kinase |
| ***gltA*** | Citrate synthase |
| ***zwf*** | Glucose-6-phosphate dehydrogenase |
| ***ppc*** | PEP carboxylase |
| ***aceE*** | Pyruvate dehydrogenase |
| ***eno*** | Enolase |
| ***pgi*** | Phosphoglucose isomerase |
| ***fba*** | Fructose-1,6-bisphosphate aldolase |

**Supplementary Figures**


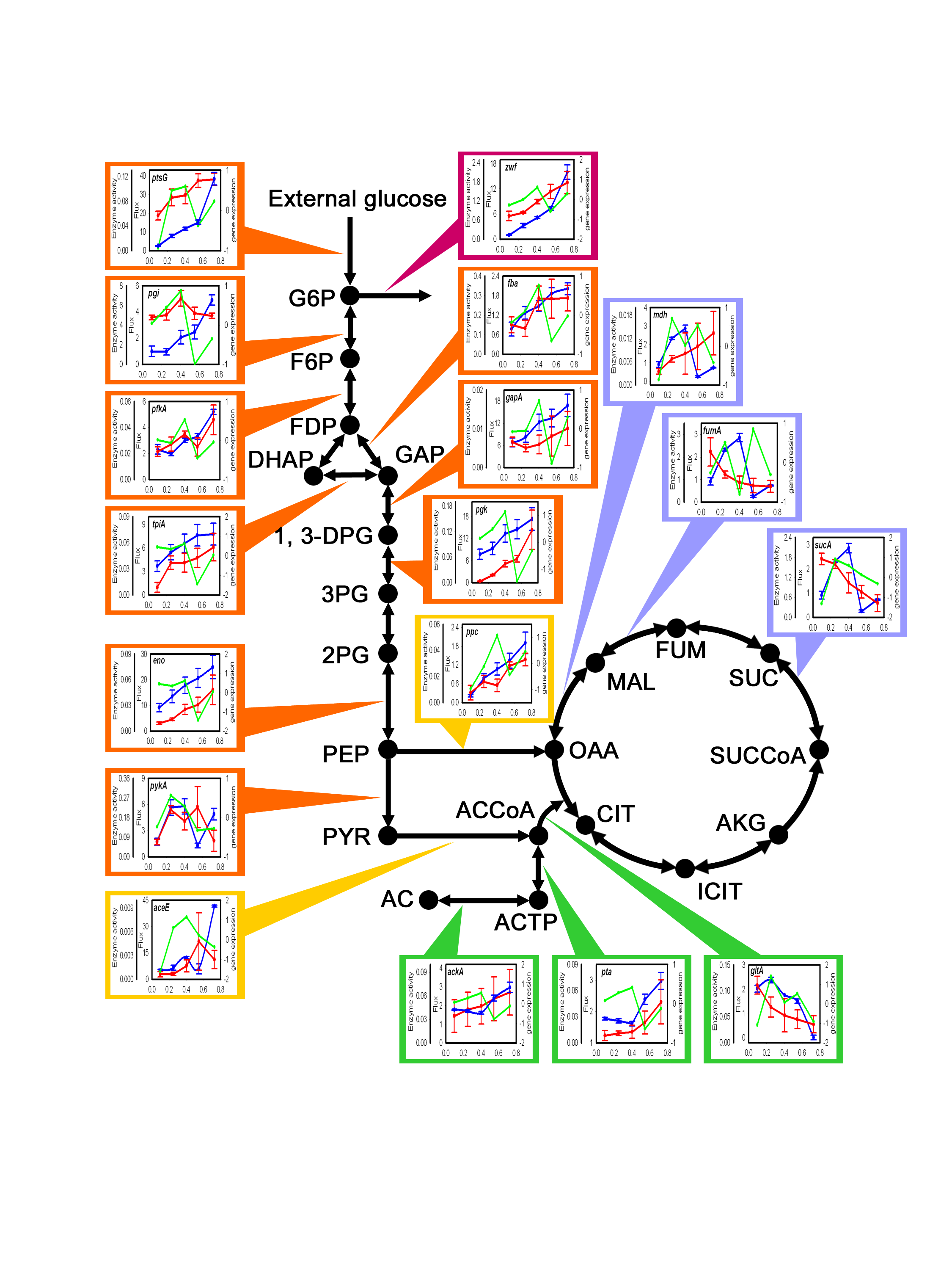


**SI Fig. 5:** Comparison of measured metabolic fluxes, enzyme activities, and relative mRNA levels: Measured flux rates (blue, mmol/h/g dry biomass), *in vitro* enzyme activities (red, U/mg protein), and gene expression levels (green, log ratio) on three separate Y-axis of selected reactions in the central metabolism of *E. coli* are shown as a function of growth/dilution rates (X-axis). All labels are as in Figure 4 in the main text. The error bars for the experimental flux and enzyme activity plots are a result of three independent measurements. For enzymes encoded by more than one gene (isozymes or enzyme complexes) we report the mRNA levels of more than one gene: *pfkA* and *pfkB*; *pykA* and *pykF*; *aceE* and *aceF*; *sucABCD*; and *fumA* and *fumC* in SI text 8 and SI Figs. 37-41.


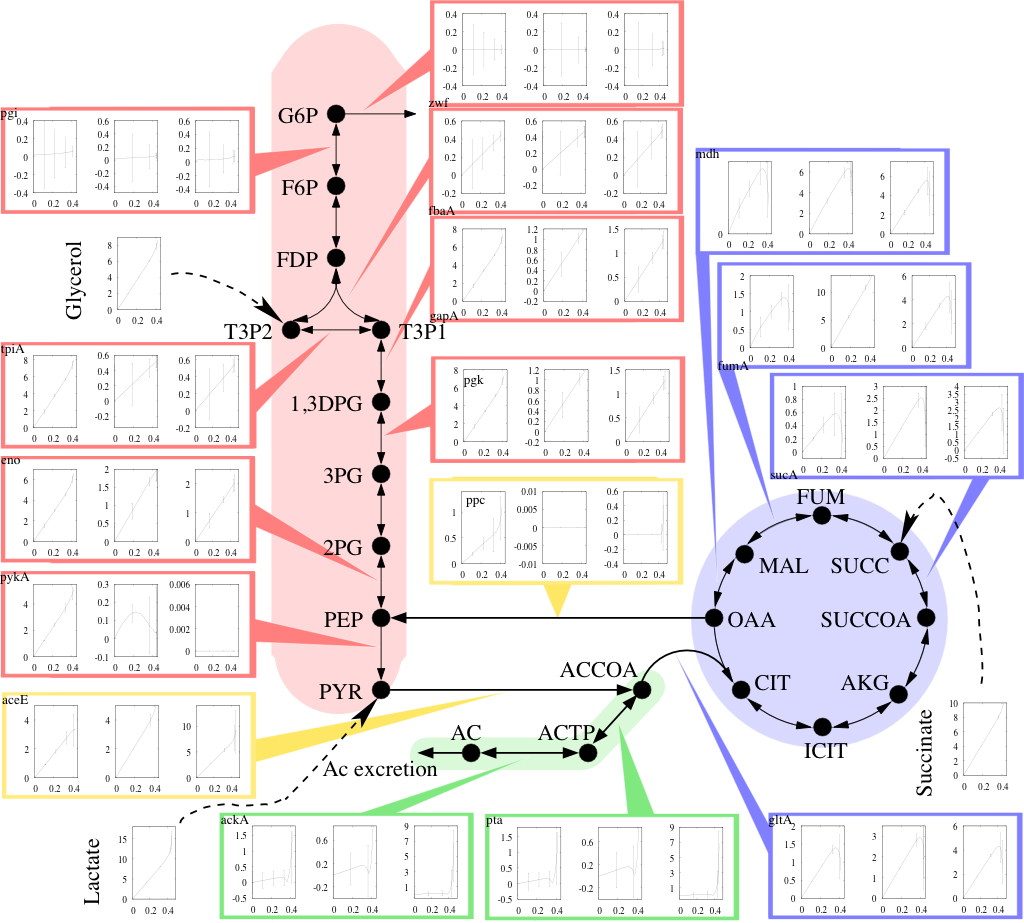


**SI Fig. 6:** Theoretical predictions of flux for various reactions in the central carbon metabolism in *E. coli* during growth on glycerol, lactate, and succinate. The panels represent the predicted fluxes in mmol/g/min as a function of the growth rate in l/h. The panels labeled with the carbon source name represent the uptake rates, while the dashed line indicates the entry point into the central metabolism.

**SI Fig. 7:** Growth (A_600nm_) and residual pO2 concentrations profile of *E. coli* MG 1655 at various dilution rates in M9 minimal medium supplemented with 0.2% glucose. Biomass samples for intracellular enzyme activity, gene expression and flux determination were harvested at the end of each major dilution rates indicated by constant A_600nm_ (optical densities) and pO_2_ concentrations in the growth medium.


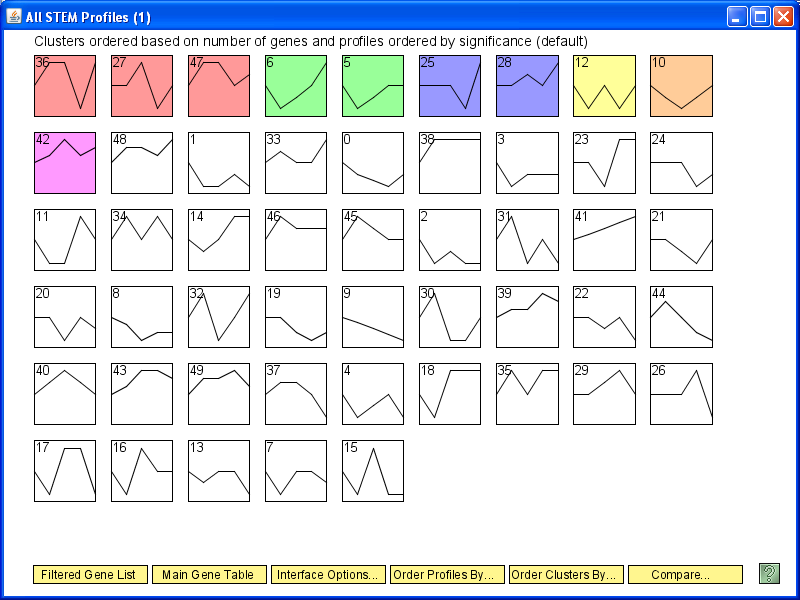


**SI Fig. 8:** Overview of STEM analysis showing all profiles considered. The profiles that are colored had a significant number of genes assigned, and similar profiles that were significant have the same color.

**
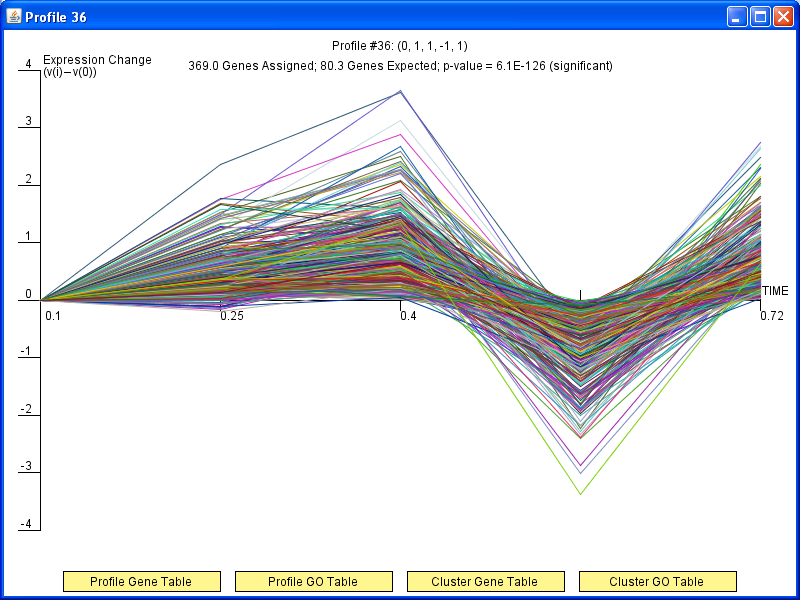

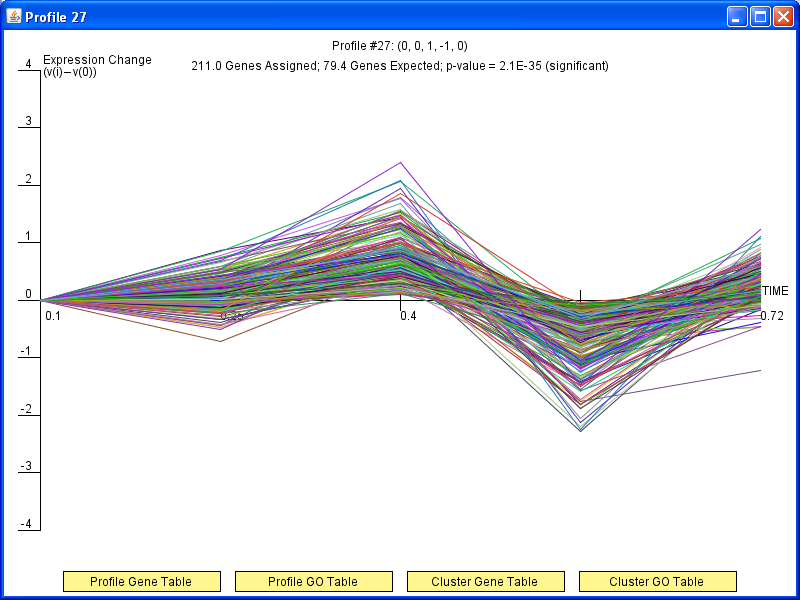

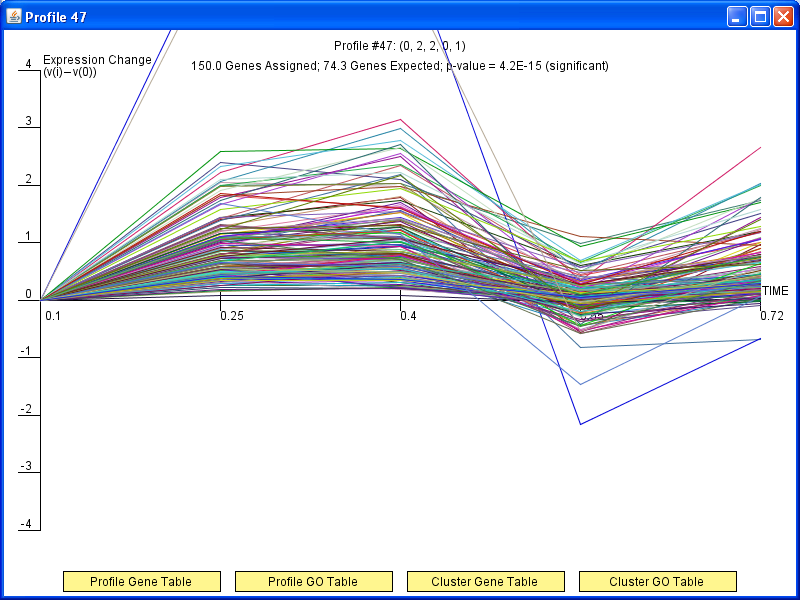
**

**
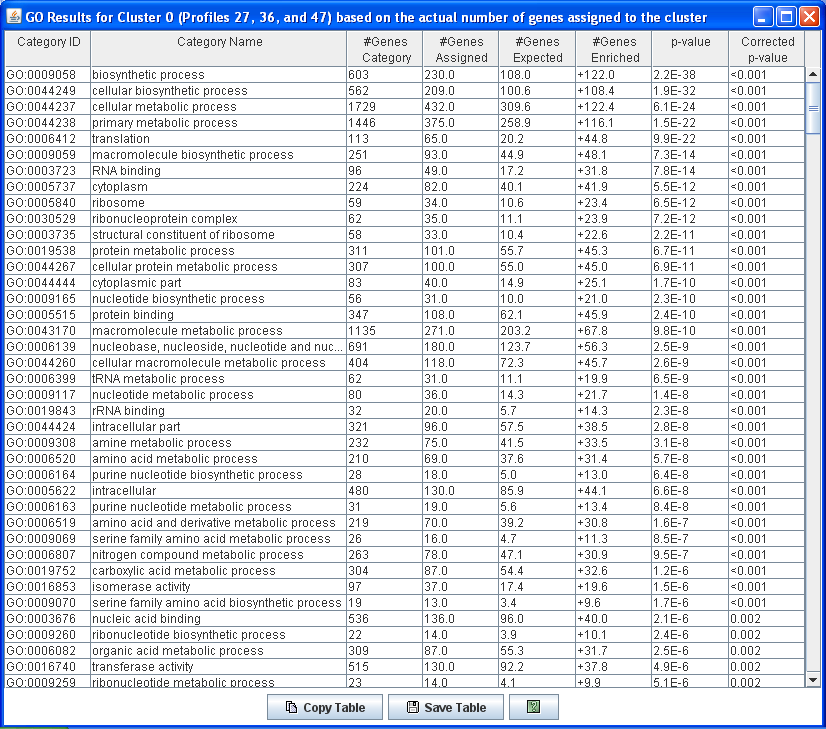
**

**SI Fig. 9:** Cluster 1 (Profiles 27, 36, and 47 from SI Fig. 8) and GO results

**
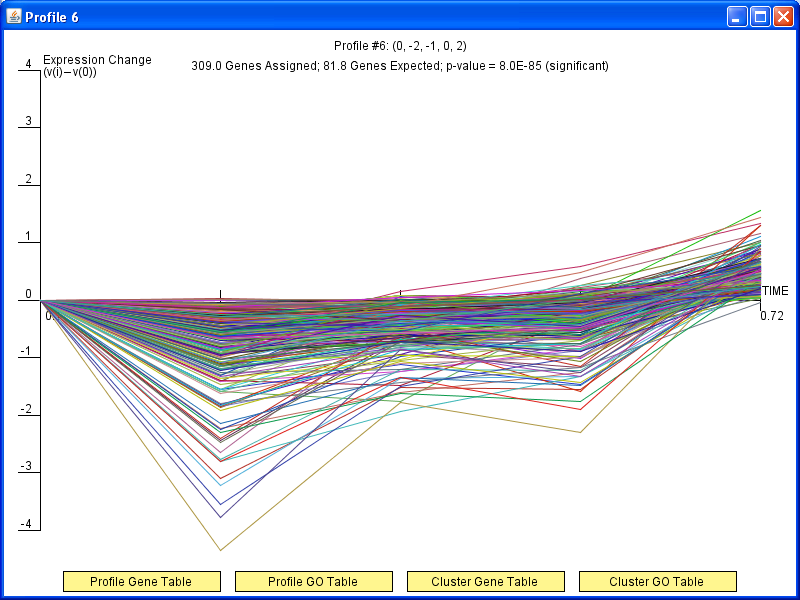

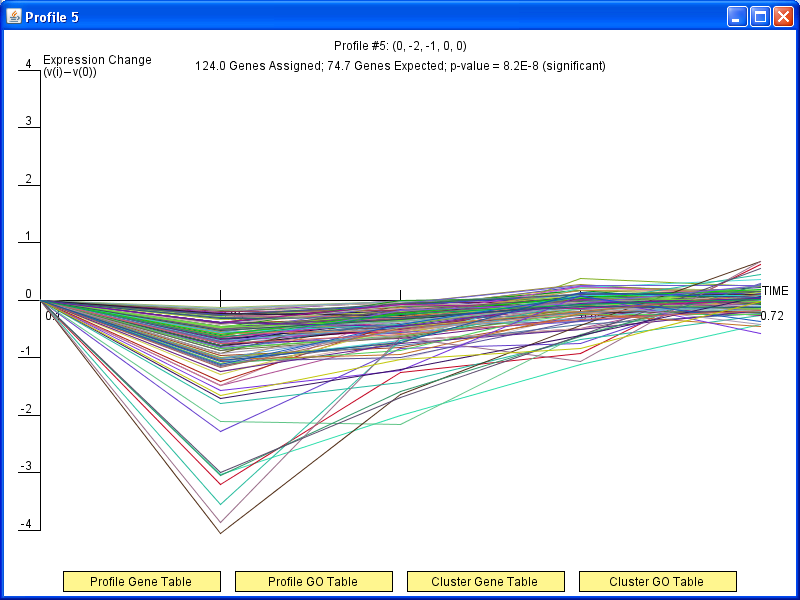
**

**
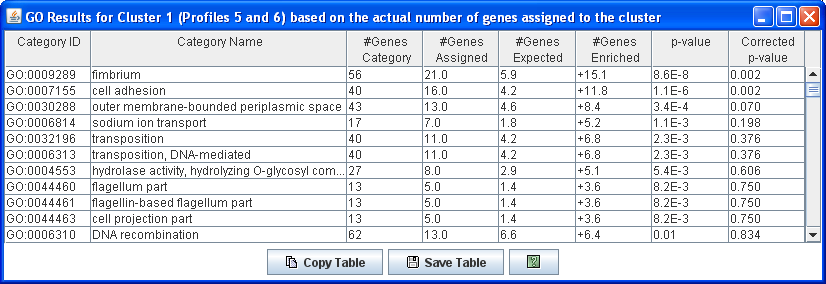
**

**SI Fig. 10:** Cluster 2 (Profiles 5 and 6 from SI Fig. 8) and GO results

**
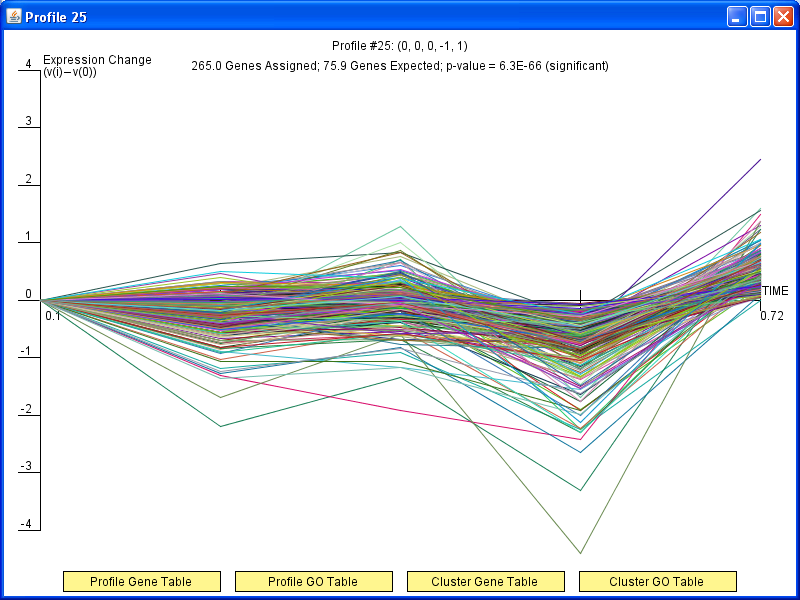

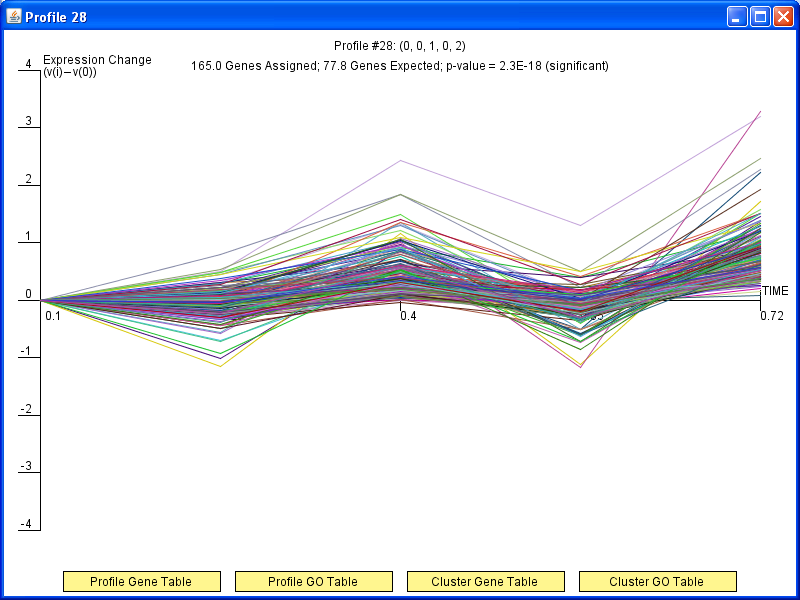
**

**
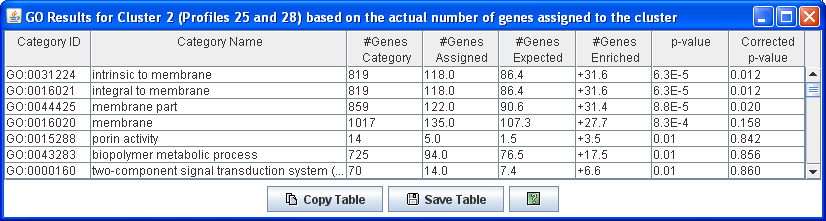
**

**SI Fig. 11:** Cluster 3 (Profiles 25 and 28 from SI Fig. 8) and GO results

**
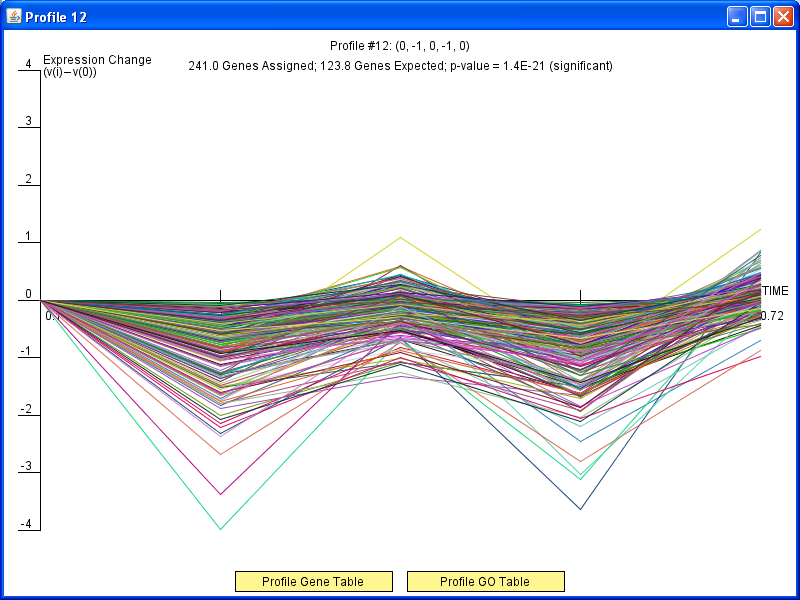
**

**
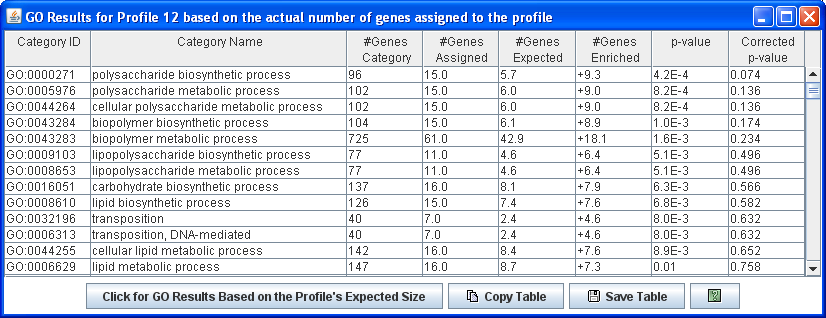
**

**SI Fig. 12:** Cluster 4 (Profile 12 from SI Fig. 8) and GO results

**
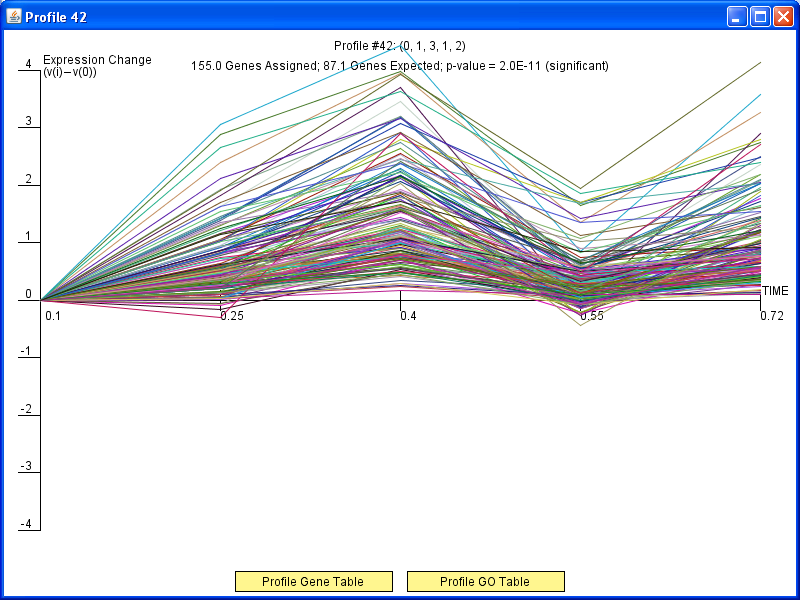
**

**
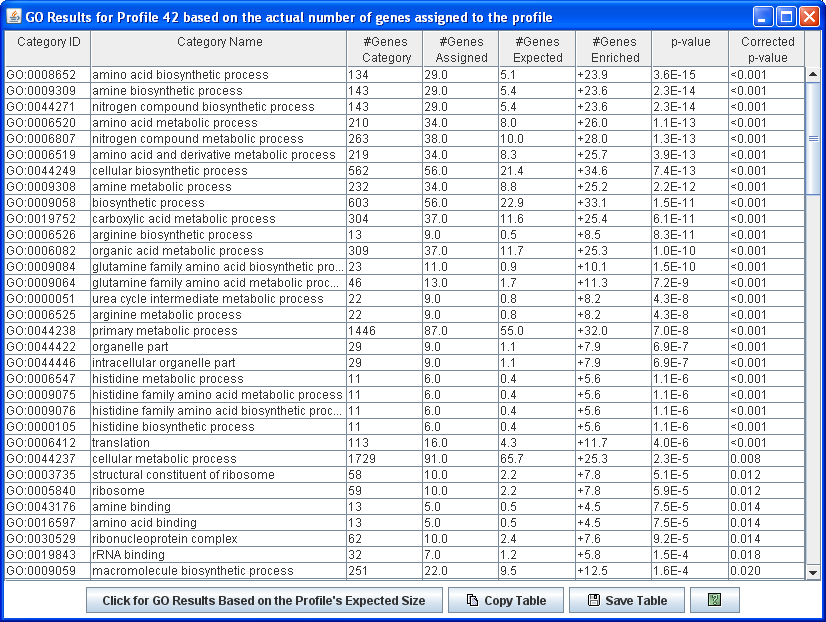
**

**SI Fig. 13:** Cluster 5 (Profile 42 from SI Fig. 8) and GO results


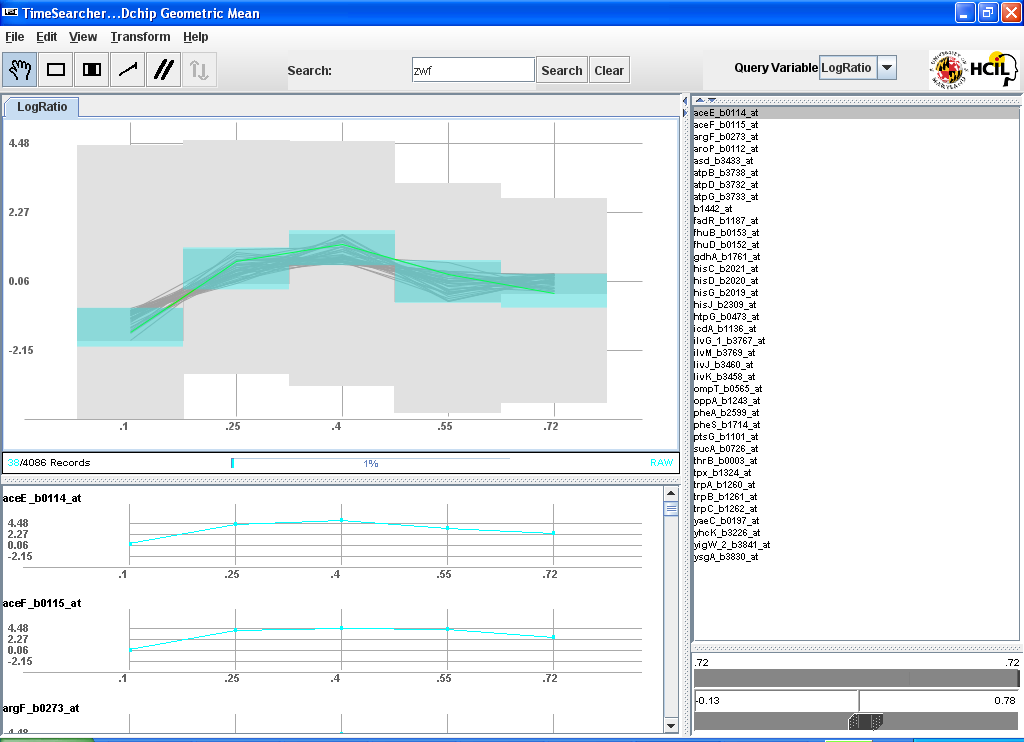


**SI Fig. 14. : *aceE***


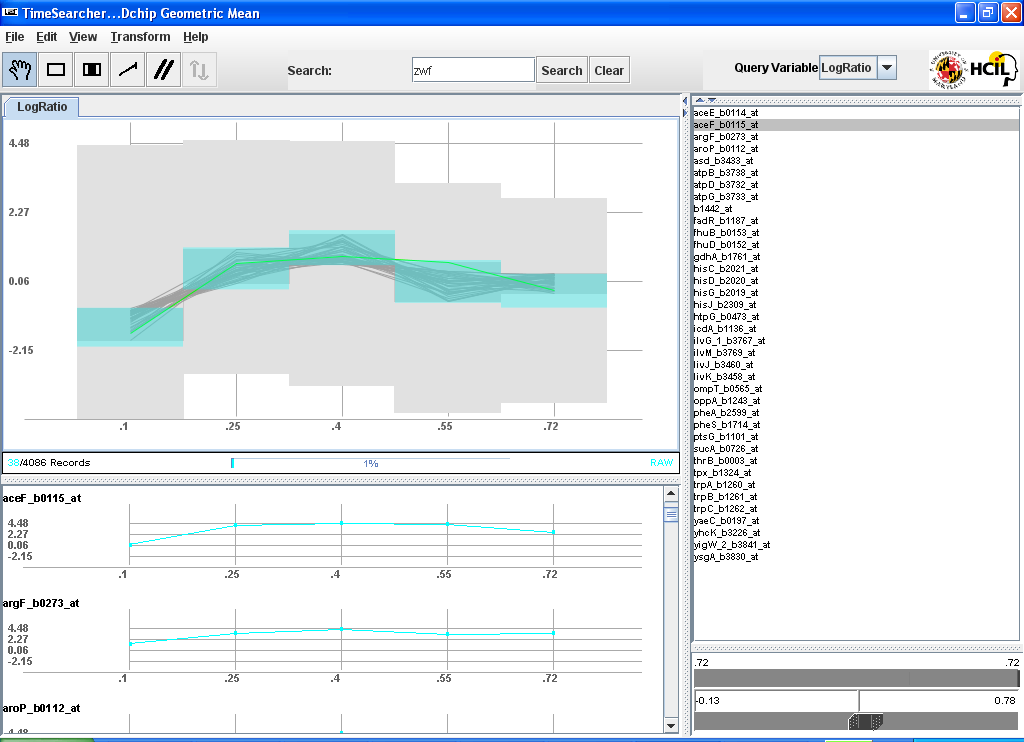


**SI Fig. 15: *aceF***


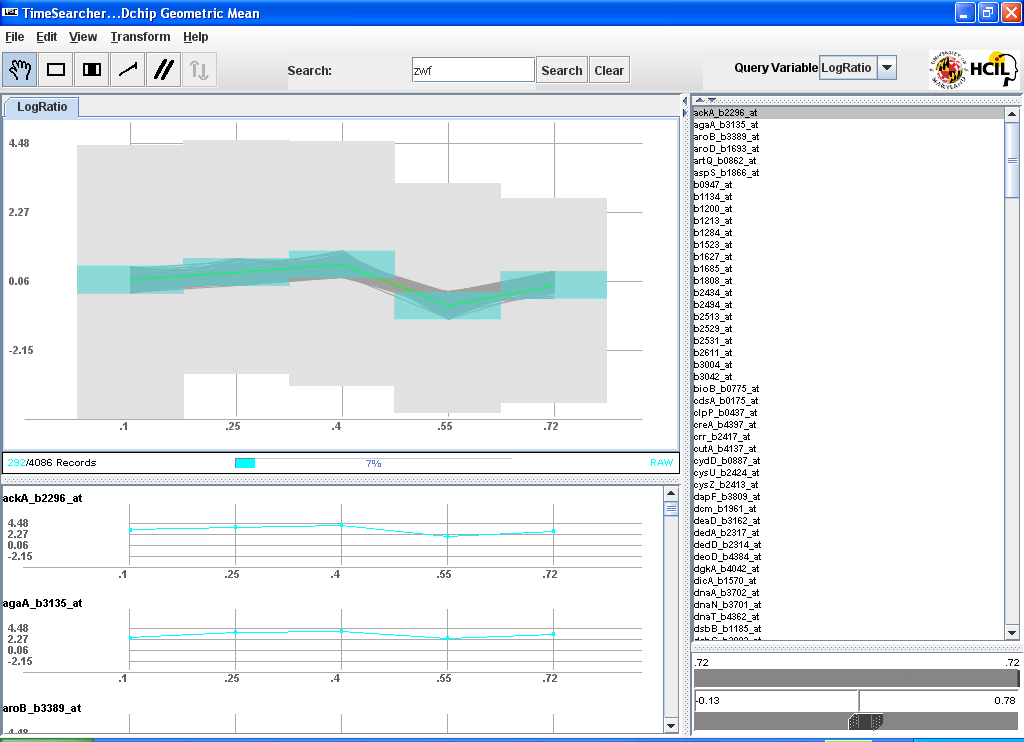


**SI Fig. 16: *ackA***


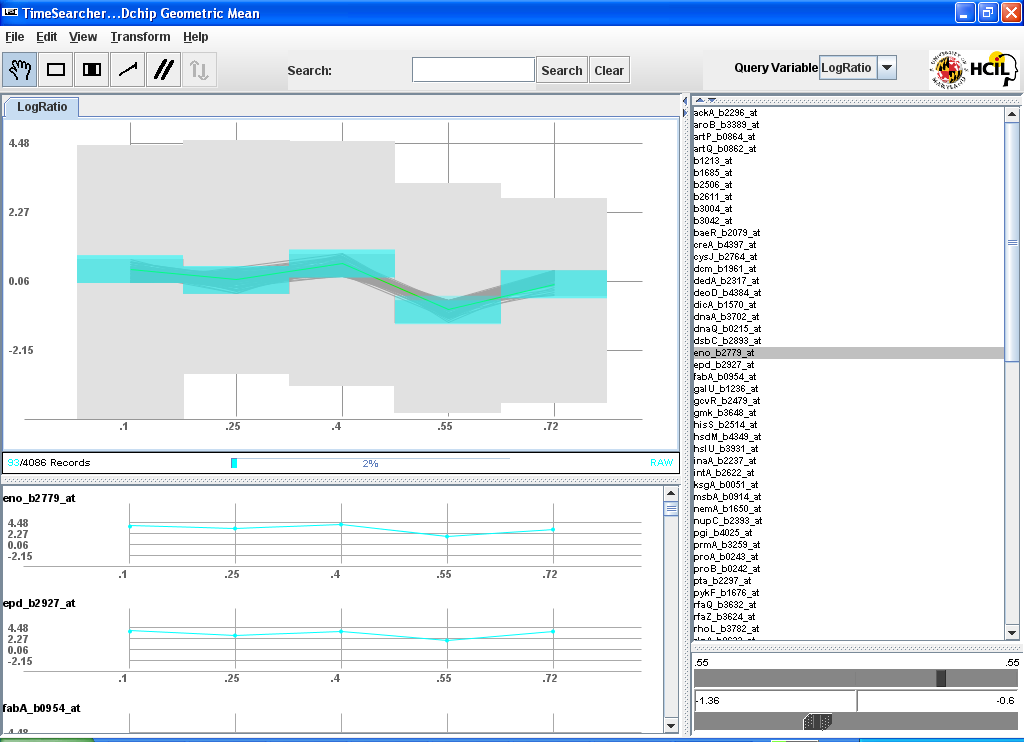


**SI Fig. 17: *eno***


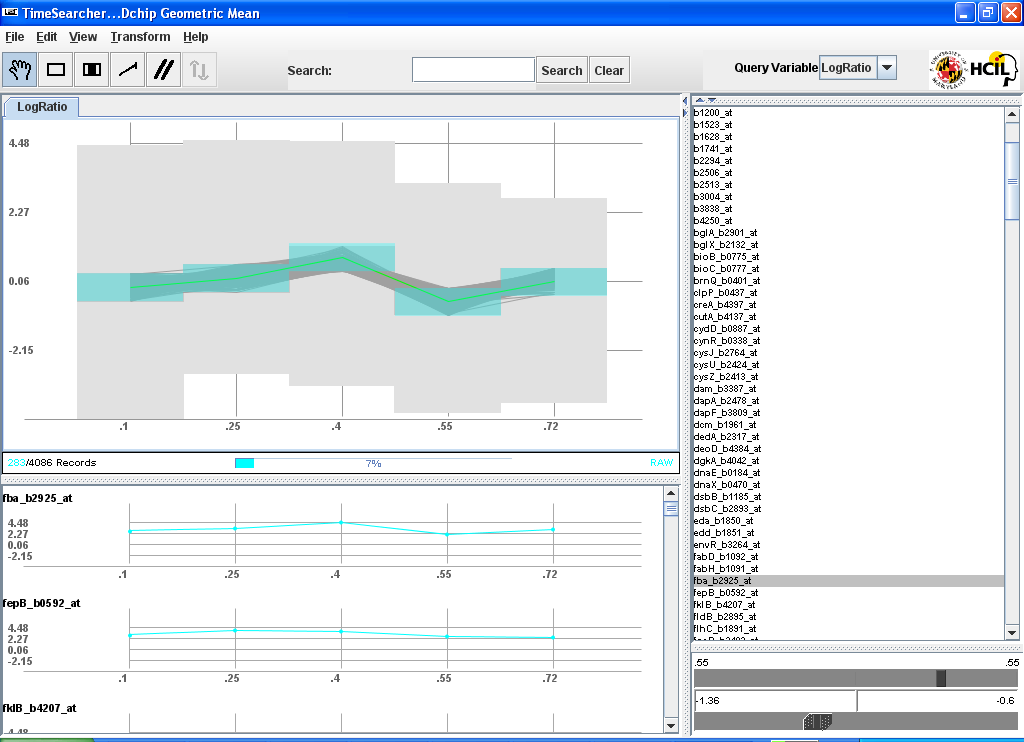
 **SI Fig. 18: *fba***


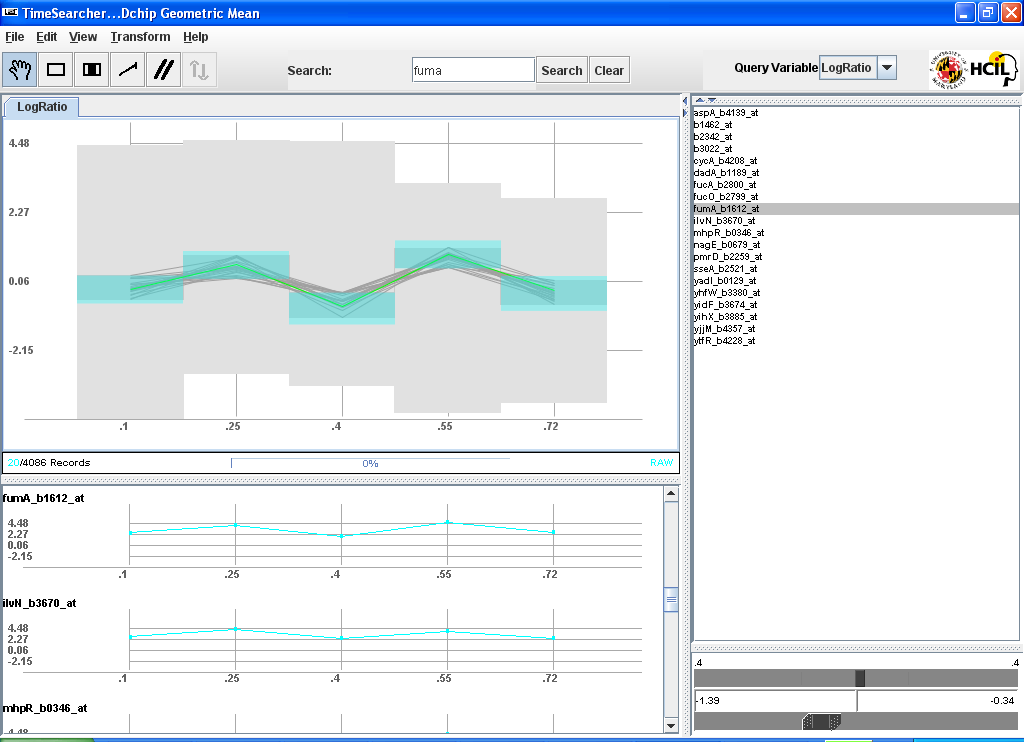


**SI Fig. 19: *fumA***


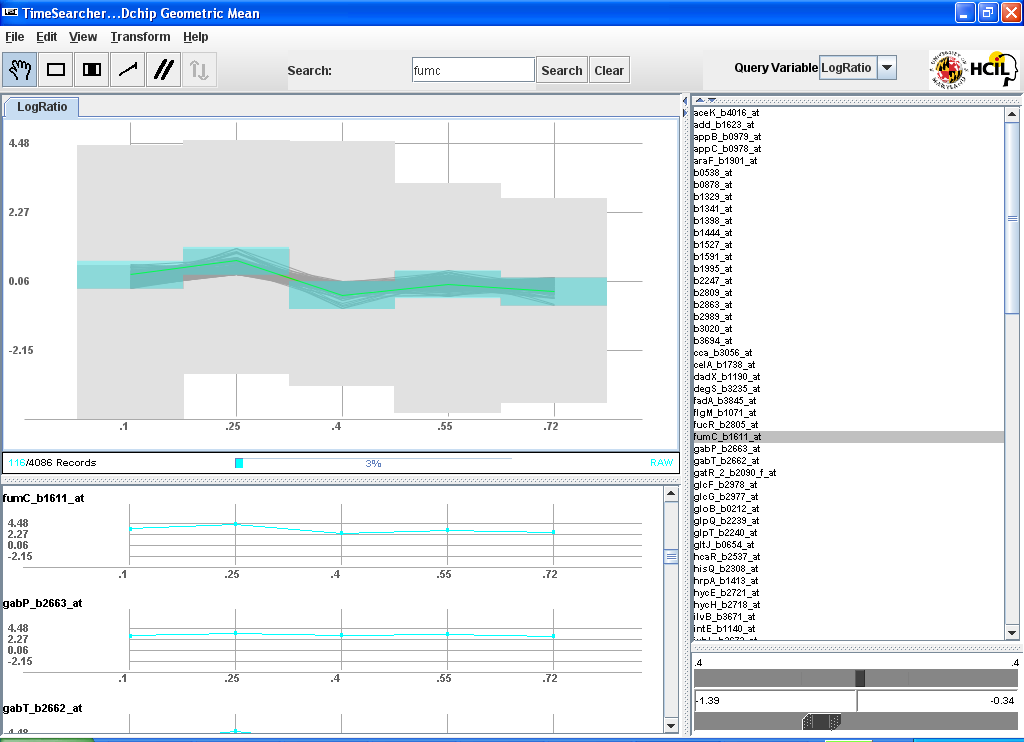


**SI Fig. 20: *fumC***

**
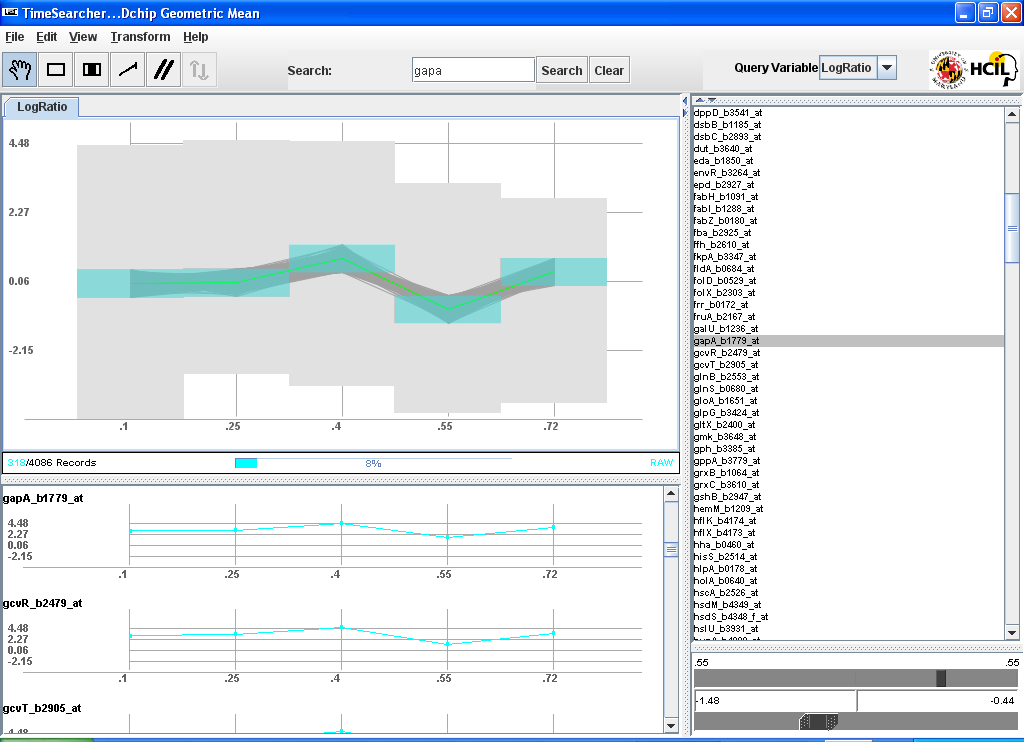
**

**SI Fig. 21: *gapA***


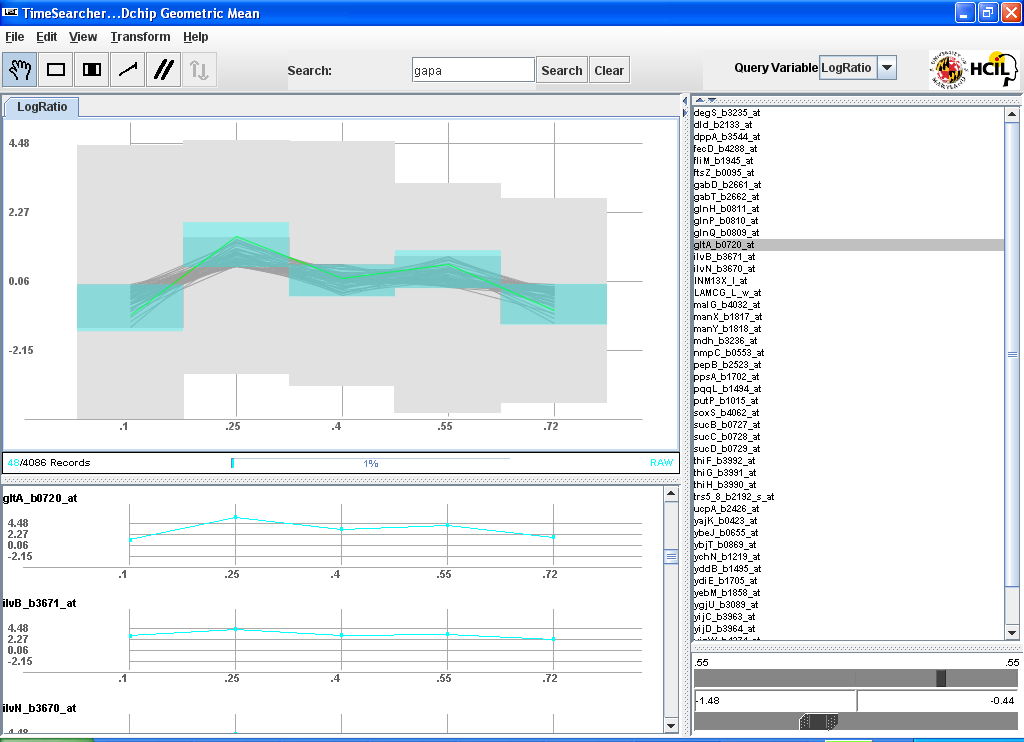


**SI Fig. 22: *gltA***

**
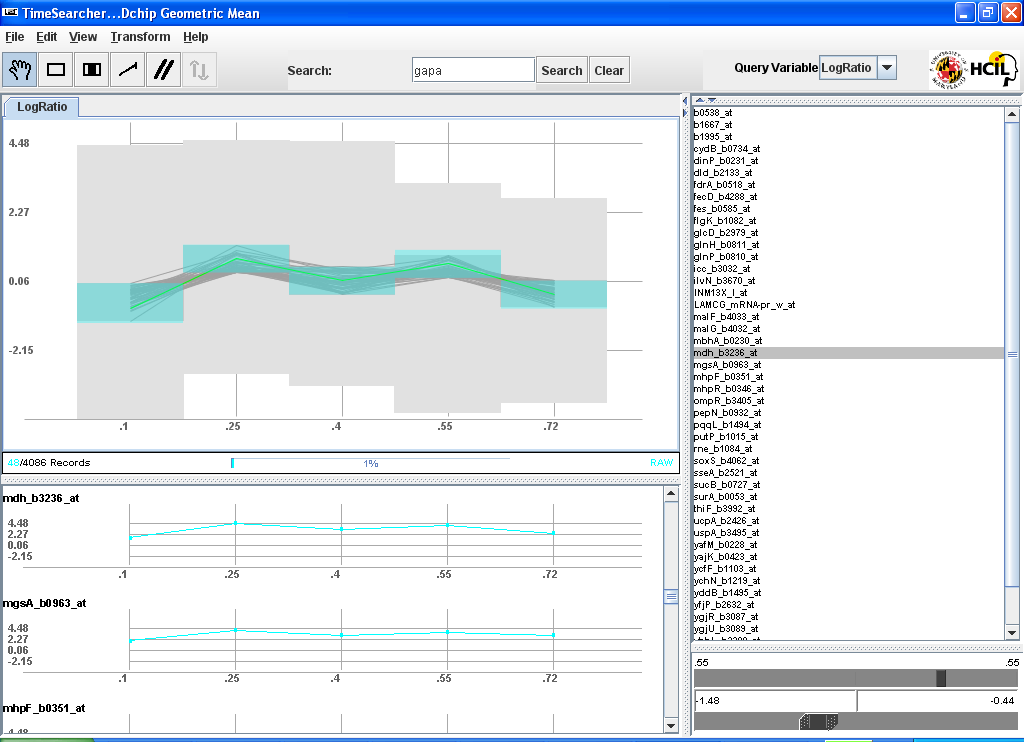
**

**SI Fig. 23: *mdh***

**
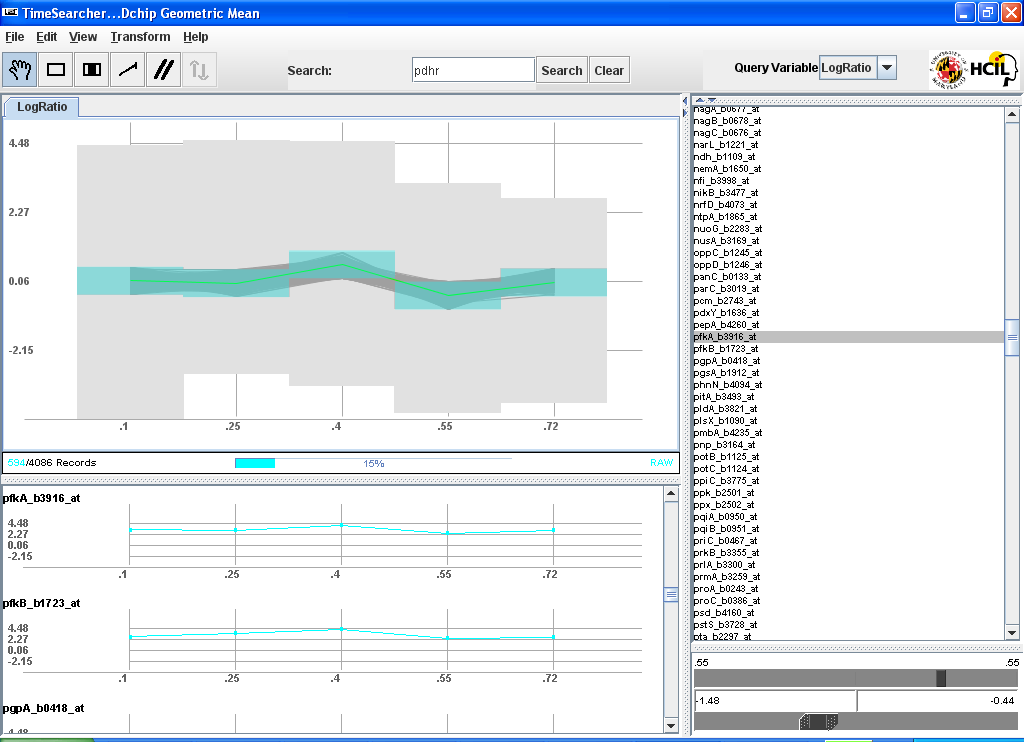
**

**SI Fig. 24: *pfkA***


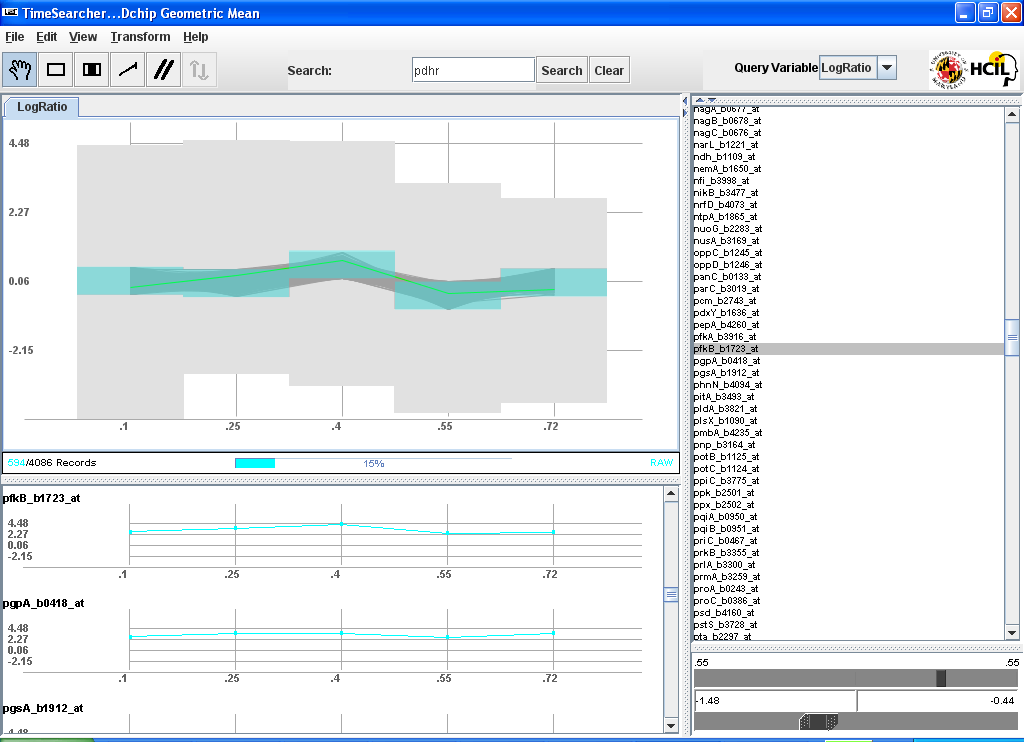


**SI Fig. 25: *pfkB***


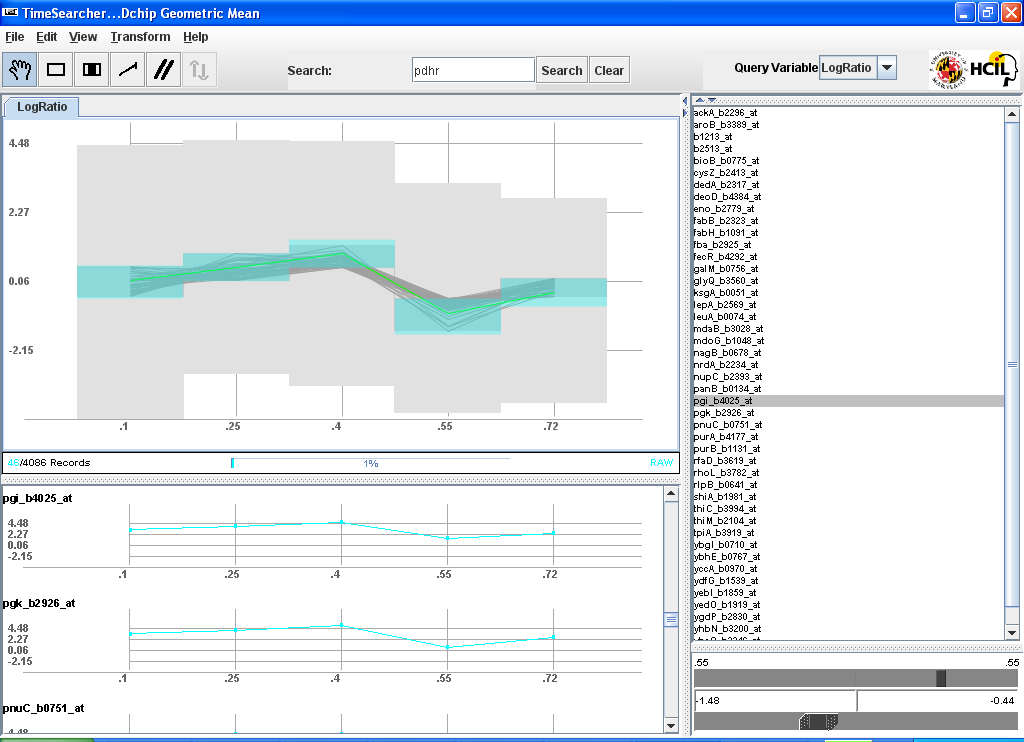


**SI Fig. 26: *pgi***


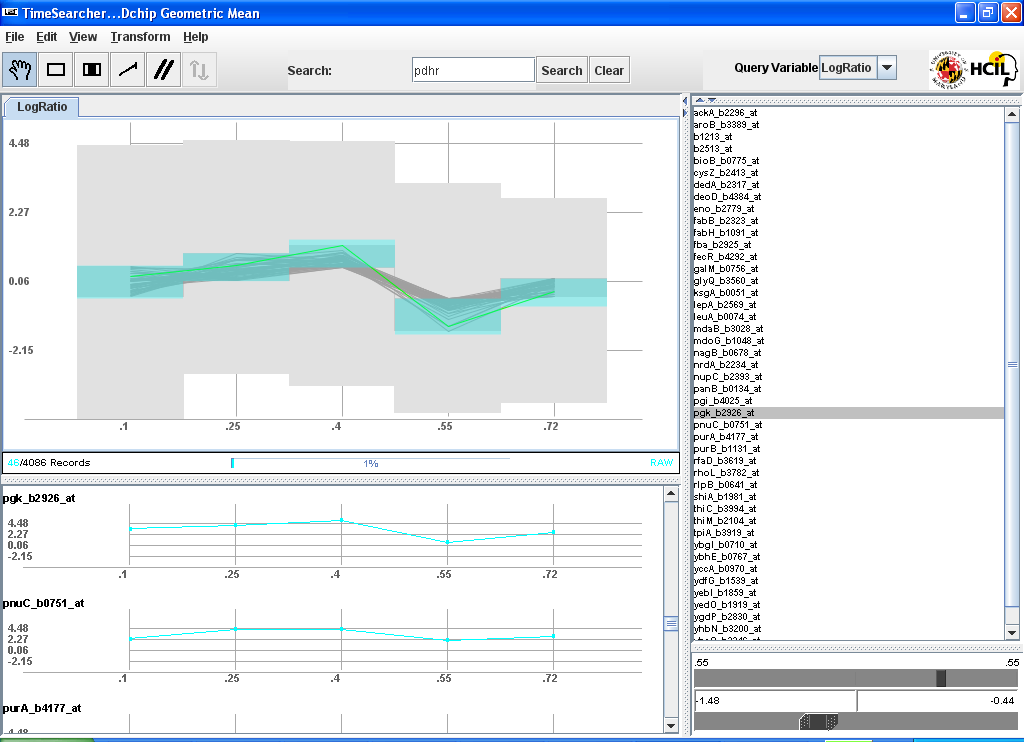


**SI Fig. 27: *pgk***

**
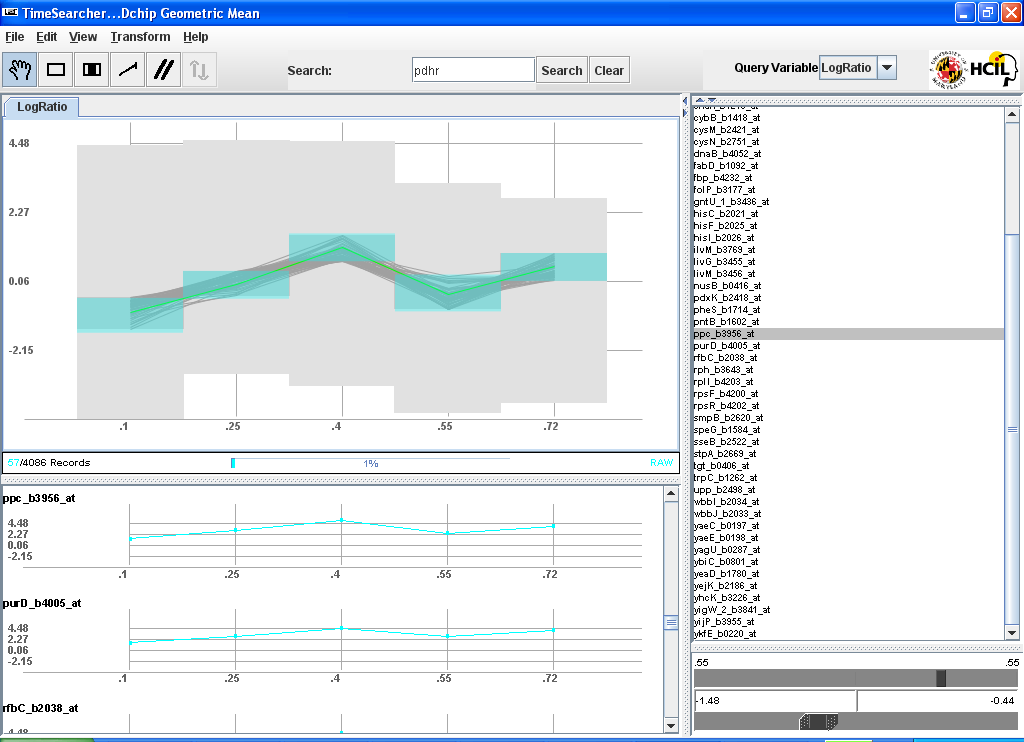
**

**SI Fig. 28: *ppc***


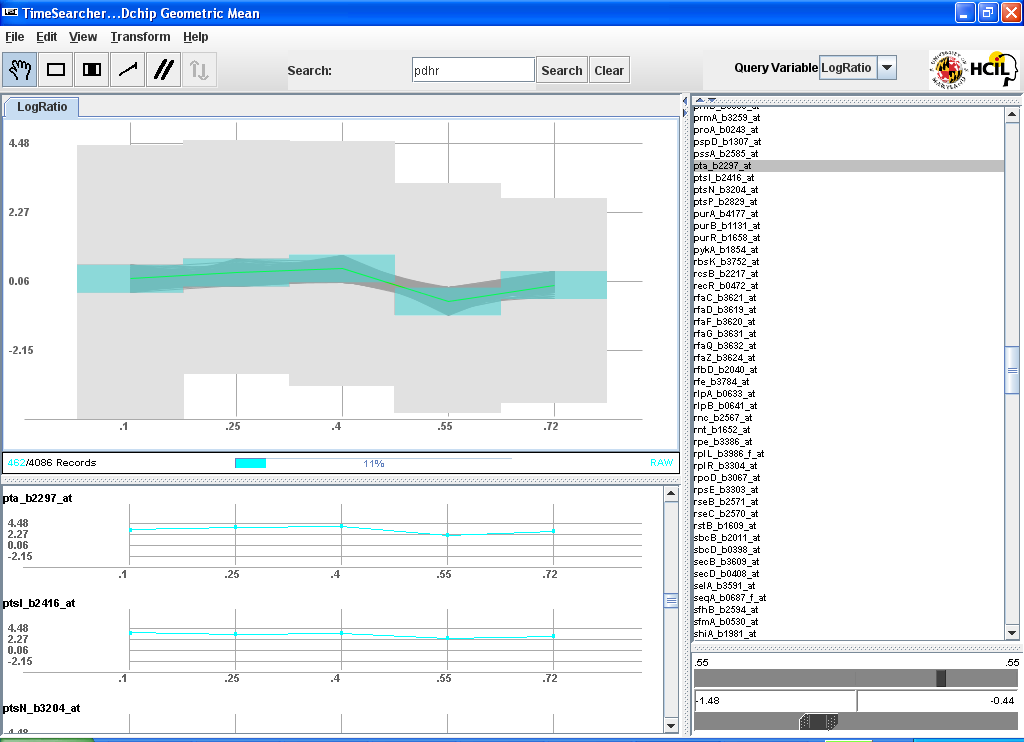


**SI Fig. 29: *pta***

**
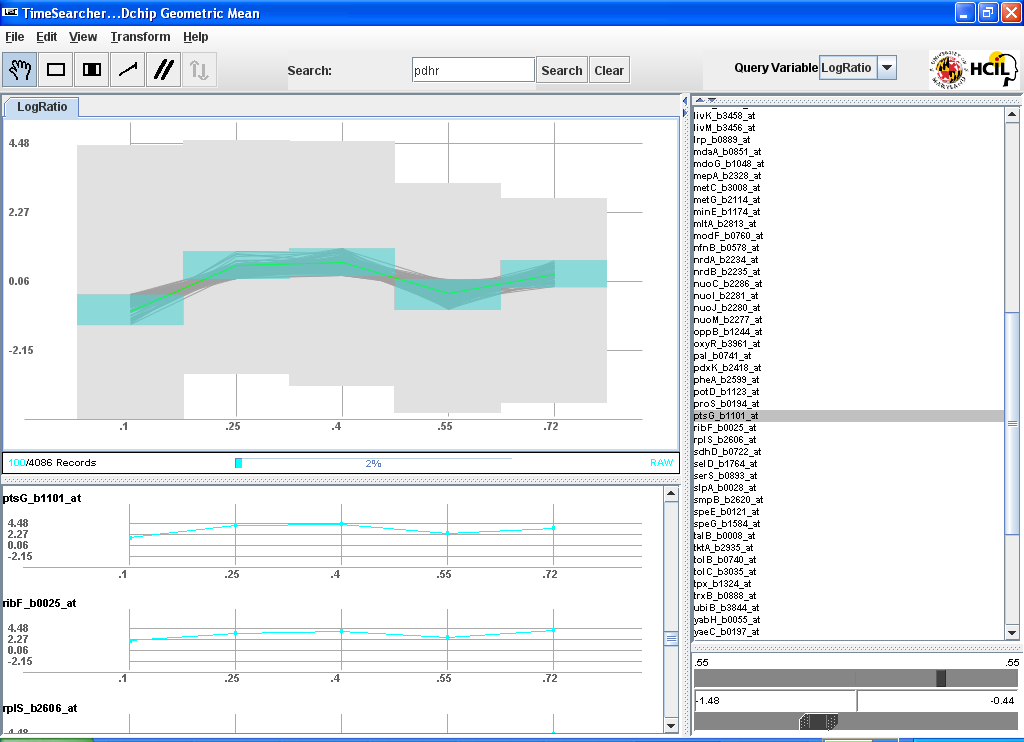
**

**SI Fig. 30: *ptsG***

**
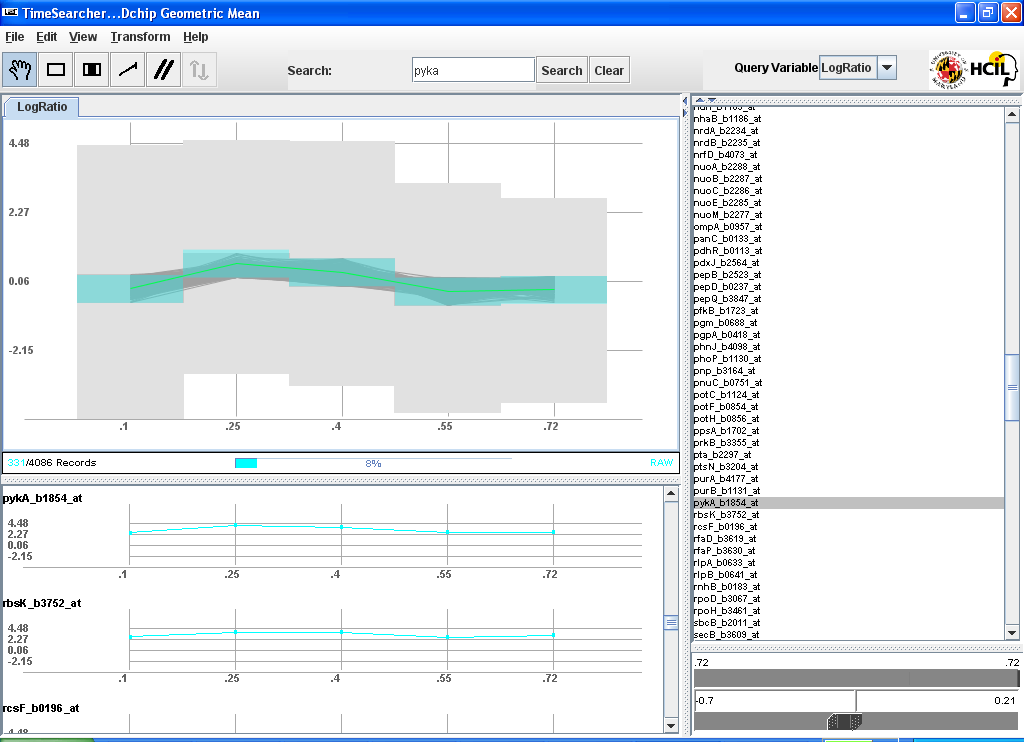
**

**SI Fig. 31: *pykA***


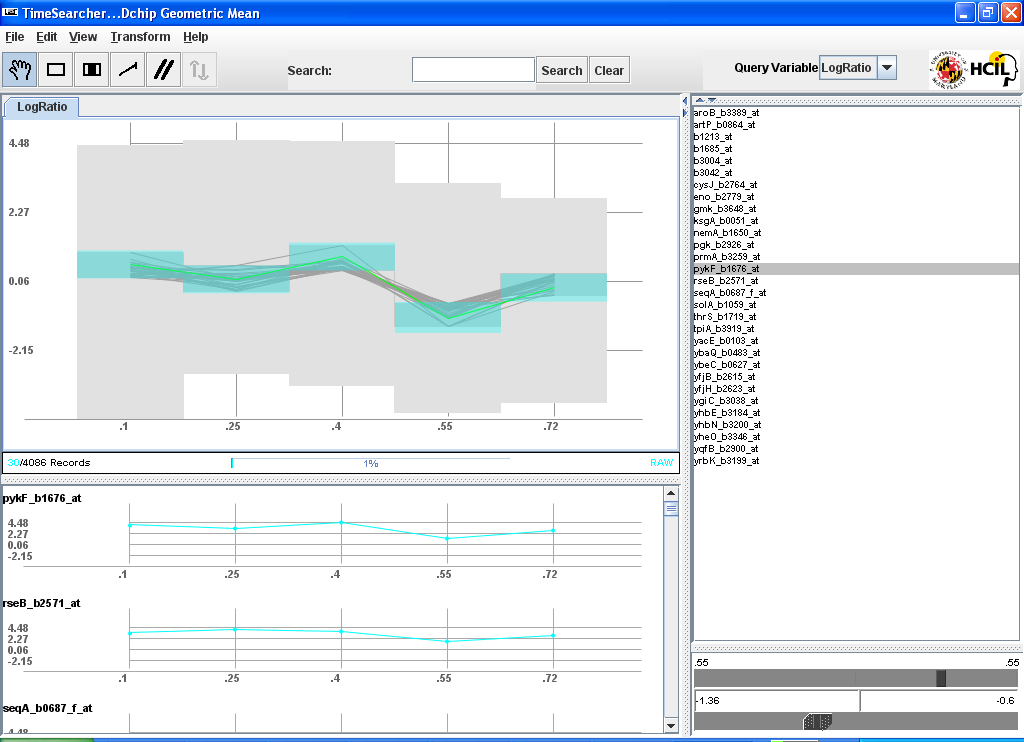


**SI Fig. 32: *pykF***

**
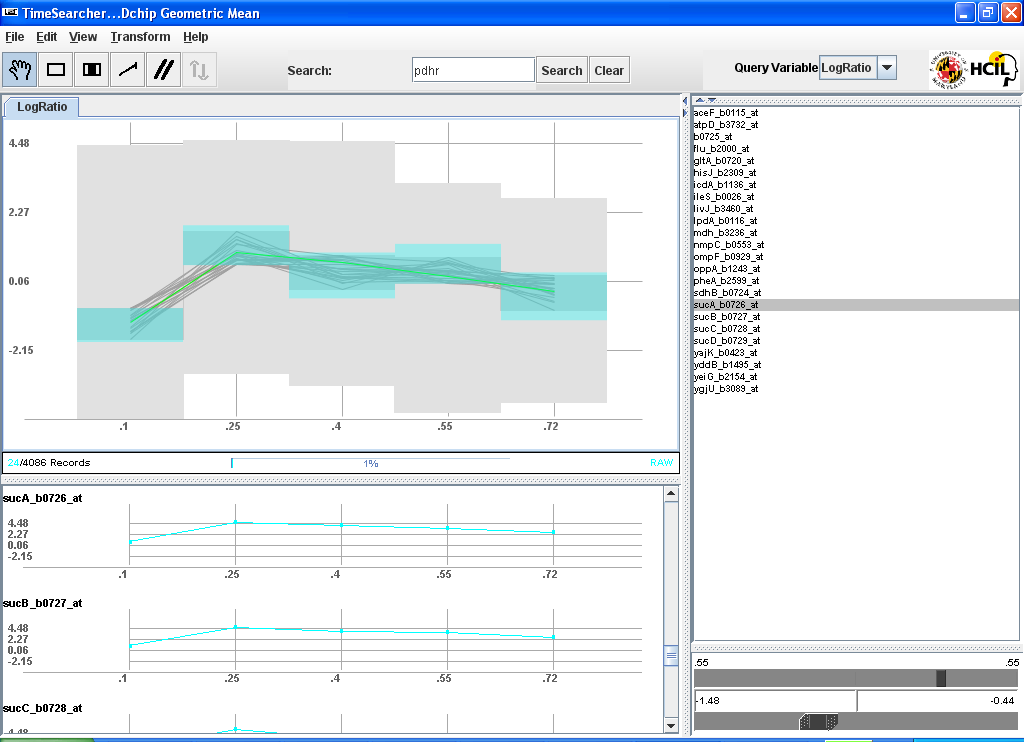
**

**SI Fig. 33: *sucA***


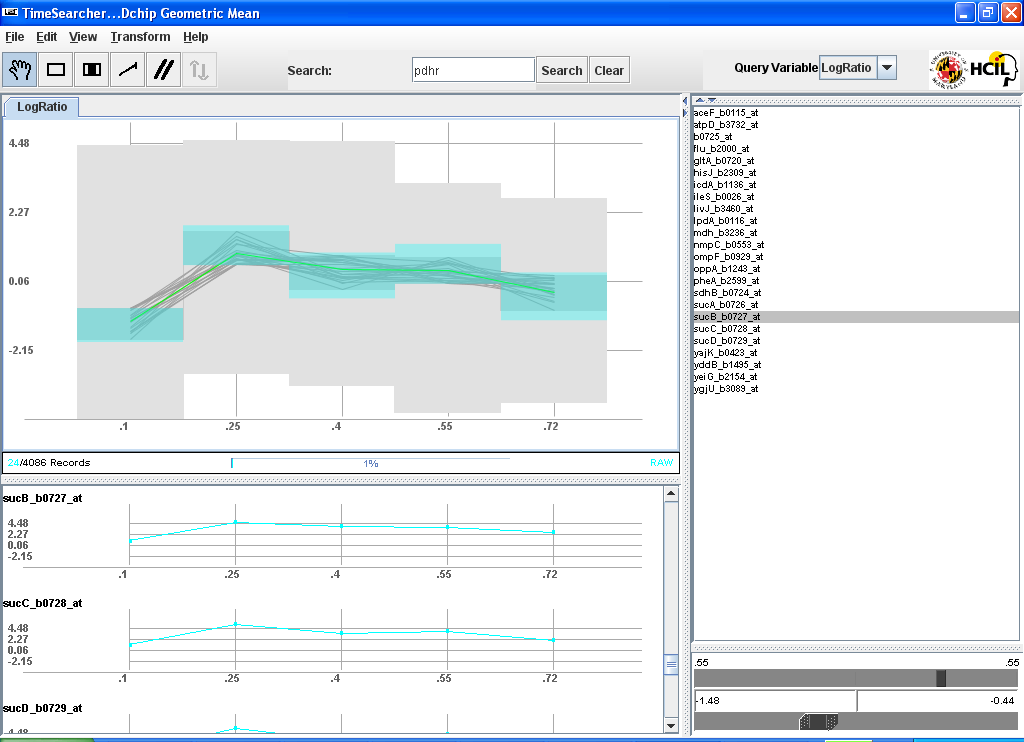


**SI Fig. 34: *sucB***


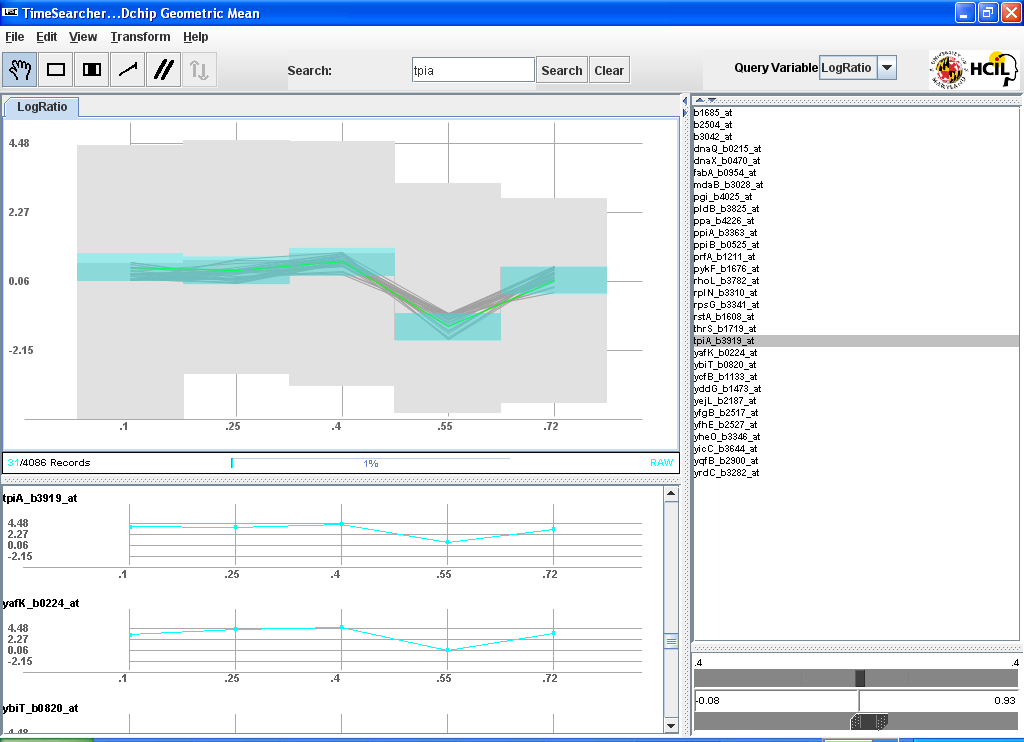


**SI Fig. 35: *tpiA***

**
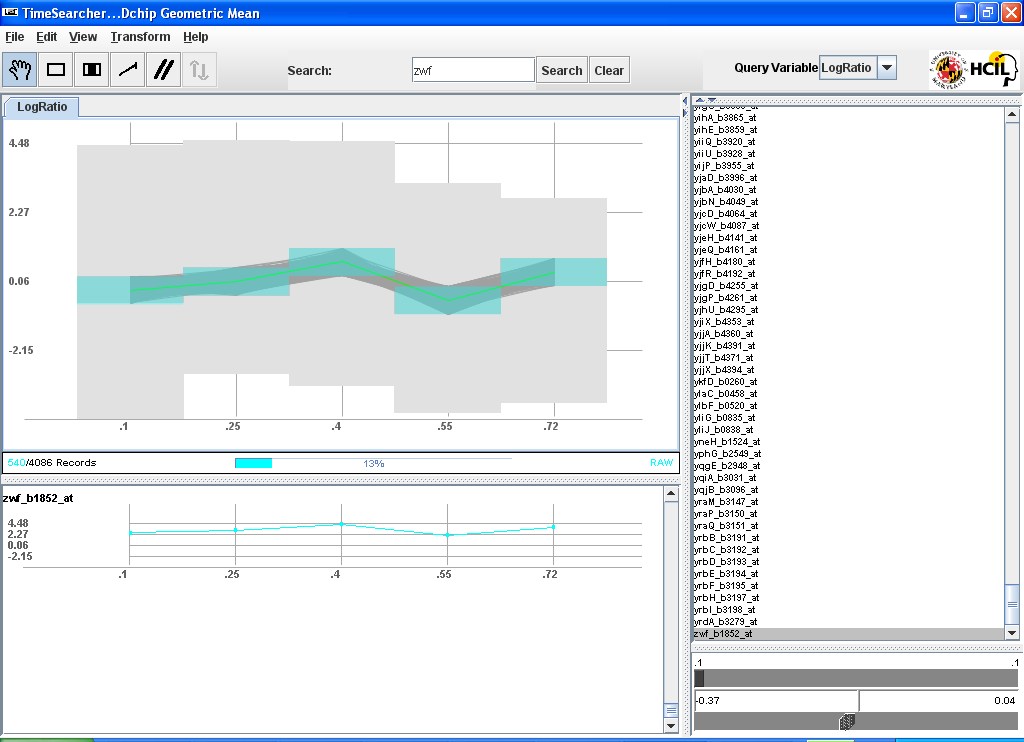
**

**SI Fig. 36: *zwf***


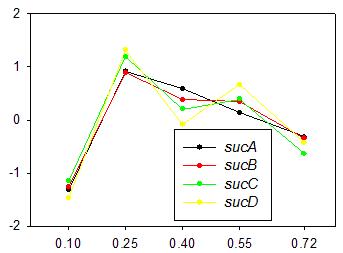


**SI Fig. 37:** *sucAB*: α-ketogluratate to Succinyl CoA; *sucCD*: reverse reaction between succinate and succinyl CoA


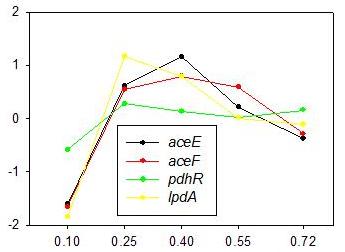


**SI Fig. 38:** Pyruvate to Acetyl-CoA


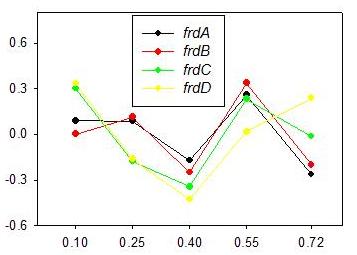


**SI Fig. 39:** Fumarate to Succinate


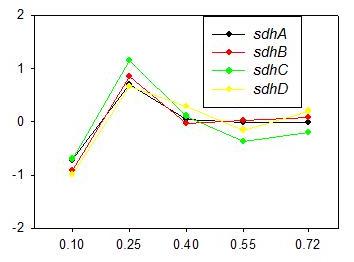


**SI Fig. 40:** Succinate to Fumarate


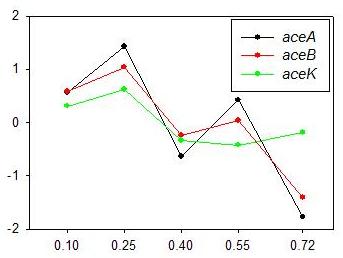


**SI Fig. 41:** Glyoxylate shunt

# References

1. Beg QK, Vazquez A, Ernst J, de Menezes MA, Bar-Joseph Z, Barabási A-L, Oltvai ZN (2007) Intracellular crowding defines the mode and sequence of substrate uptake by *Escherichia coli* and constrains its metabolic activity. Proc Natl Acad Sci USA 104: 12663-12668.
2. Boros LG, Cascante M, Lee WN (2002) Metabolic profiling of cell growth and death in cancer: applications in drug discovery. Drug Discov Today 7: 364 - 372
3. Ernst, J and Bar-Joseph, Z. STEM: a tool for the analysis of short time series gene expression data. *BMC Bioinformatics* 2006, 7:191.
4. Gottschalk S, Anderson N, Miljus J, Eckhardt SG, Serkova NJ (2004) Imatinib (STI571)-mediated changes in glucose metabolism in human leukemia BCR-ABL positive cells. Clin Cancer Res 10: 6661-6668.
5. Hochheiser, H., Baehrecke, E.H., Mount, S.M., and Shneiderman, B. Dynamic Querying for Pattern Identification in Microarray and Genomic Data. In *Proceedings IEEE Multimedia Conference and Expo* 2003, (IEEE, Piscataway, NJ), Vol. 3, pp. III-453-III-456
6. Lee WN, Boros LG, Puigjaner J, Bassilian S, Lim S, Cascante M (1998a) Mass isotopomer study of the transketolase-transaldolase pathways of the pentose cycle with [1,2-^13^C2]glucose. Am J Physiol 274: E843 - E851
7. Lee WN, Byerley LO, Bassilian S, Ajie HO, Clark I, Edmond J (1995) Isotopomer study of lipogenesis in human hepatoma cells in culture: contribution of carbon and hydrogen atoms from glucose. Anal Biochem 226: 100 - 112
8. Lee WN, Edmond J, Bassilian S, Morrow JW (1996) Mass isotopomer study of glutamine oxidation and synthesis in primary culture of astrocytes. Developmental Neuroscience 18: 469 – 477
9. Lee WN, Lim S, Bassilian S, Bergner EA, Edmond J (1998b) Fatty acid cycling in human hepatoma cells and the effects of troglitazone. J Biol Chem 273: 20929 - 20934
10. Leimer KR, Rice RH, Gehrke CW (1977) Complete mass spectra of N-TAB esters of amino acids. J Chromatography 141: 121 - 144
11. Li, C and Wong W. Model-based analysis of oligonucleotide arrays: model validation, design issues and standard error application. *Genome Biology* 2001, 2: research0032.1-0032.11.
12. Peng, L. & Shimizu, K. (2003) Global metabolic regulation analysis for *Escherichia coli* K12 based on protein expression by 2-dimensional electrophoresis and enzyme activity measurement. Appl Microbiol Biotechnol 61, 163-78.
13. Serkova N, Bendrick-Peart J, Alexander B, Tissot van Patot MC (2003) Metabolite concentrations in human term placentae and their changes due to delayed collection after delivery. Placenta 24: 227-235.
14. Serkova N, Fuller TF, Klawitter J, Freise CE, Niemann CU (2005) ^1^H-NMR-based metabolic signatures of mild and severe ischemia/ reperfusion injury in rat kidney transplants. Kidney Int 67: 1142-1151.
15. Van der Werf, M. J., Guettler, M. V., Jain, M. K. & Zeikus, J. G. (1997) Environmental and physiological factors affecting the succinate product ratio during carbohydrate fermentation by *Actinobacillus* sp. 130Z. Arch Microbiol 167, 332-342.
16. Xu J, Lee WN, Xiao G, Trujillo C, Chang V, Blanco L, Hernandez F, Chung B, Makabi S, Ahmed S, Bassilian S, Saad M, Kurland IJ.( 2003) Determination of a glucose-dependent futile recycling rate constant from an intraperitoneal glucose tolerance test. Anal Biochem. 315: 238-246.
17. Yang L, Kasumov T, Yu L, Jobbins KA, David F, Previs SF, Kelleher JK, Brunengraber H. Metabolomic assays of the concentration and mass isotopomer distribution of gluconeogenic and citric acid cycle intermediates. Metabolomics 2: 85-94.
18. Zhao, J., Baba, T., Mori, H. & Shimizu, K. (2004) Global metabolic response of *Escherichia coli* to *gnd* or *zwf* gene-knockout, based on ^13^C-labeling experiments and the measurement of enzyme activities. Appl Microbiol Biotechnol 64, 91-98.
